# From Academic Integrity to Professional Formation in Generative AI–Supported Nursing Education: Integrative Review

**DOI:** 10.2196/99284

**Published:** 2026-10-30

**Authors:** Peter Hartin, Paul Baretta

**Affiliations:** 1 College of Healthcare Sciences, James Cook University, 1 James Cook Drive, Douglas, Queensland, 4811, Australia, 61 0404225612

**Keywords:** AI, generative AI, nursing education, professional formation, academic integrity, clinical judgment, algorithmic practice, student identity

## Abstract

**Background:**

The rapid integration of generative AI (GenAI) into nursing education has prompted widespread concern about academic integrity and assessment validity. However, this dominant discourse may obscure a more fundamental challenge: preparing nursing students for clinical practice environments where AI already mediates triage, diagnostics, documentation, and clinical decision-making.

**Objective:**

This integrative review examines how GenAI is conceptualized within the nursing education literature and investigates the extent to which current discourse addresses professional formation rather than academic compliance.

**Methods:**

Following Whittemore and Knafl’s integrative review methodology, a systematic search of PubMed, Google Scholar, and SciSpace (Typeset) was conducted for literature published between January 2022 and February 2026. Two reviewers independently screened 456 records, with 119 papers meeting the inclusion criteria. The methodological quality of empirical studies was assessed using the Mixed Methods Appraisal Tool (MMAT). Corpus-level descriptive mapping quantified dominant themes and conceptual patterns, while an interpretive synthesis examined how GenAI was framed and the extent to which professional formation elements, including identity, moral agency, and clinical judgment, were addressed.

**Results:**

Of the 119 unique studies analyzed, 84% (n=100) predominantly framed GenAI as a content-generation tool and academic integrity threat. Only 35.3% (n=42) conceptualized AI as a pedagogical tool, and a critical gap was identified in professional formation: identity formation was addressed in only 10% (n=12) of papers and moral agency in just 5% (n=6). Explicit acknowledgment that AI already routinely mediates frontline clinical decisions (eg, sepsis alerts and fall risk algorithms) in current practice settings was notably absent. The discourse emphasizes governance and faculty literacy, but sustained curricula addressing nurse identity in AI-mediated care remain underdeveloped.

**Conclusions:**

Current nursing education discourse predominantly frames AI as a writing tool and an academic integrity concern, failing to prepare students for the algorithmic practice environments they will inhabit. Nursing education must reframe AI as a professional formation issue, explicitly addressing how algorithms reshape clinical judgment, moral agency, and professional identity.

## Introduction

The rapid emergence of generative AI (GenAI), particularly large language models such as ChatGPT (OpenAI), has prompted one of the most significant pedagogical disruptions experienced by higher education in recent decades. Since ChatGPT became publicly available in late 2022, nurse educators have grappled with questions surrounding academic integrity, assessment validity, authorship, and the appropriate boundaries of AI use in student learning [1,2]. These concerns have generated a rapidly expanding body of literature examining the educational implications of GenAI and have prompted widespread institutional responses, including revised assessment practices, AI policies, and faculty development initiatives [3-5].

Alongside these educational developments, health care itself is undergoing rapid technological transformation. AI is increasingly embedded within contemporary clinical practice through technologies such as clinical decision support systems, predictive analytics, automated documentation, risk stratification algorithms, and workflow optimization tools. Unlike GenAI, which generates novel content in response to user prompts, clinical decision support systems primarily support clinicians’ decision-making using predefined rules, predictive models, or machine learning algorithms embedded within health care workflows [6,7]. Consequently, graduates entering the nursing workforce will not simply encounter AI as an educational technology but will practice within health care environments where algorithmic systems increasingly influence clinical reasoning, decision-making, and patient care. Preparing nurses for this reality requires educational approaches that extend beyond managing AI use during assessment to considering how future practitioners develop professional judgment, ethical reasoning, and accountability within algorithmically mediated practice.

Recent reviews have made important contributions to understanding GenAI in nursing education by examining issues such as academic integrity, ethical considerations, educational applications, faculty preparedness, student perceptions, and pedagogical innovation [3,8-11]. Collectively, this work has established a valuable evidence base describing how GenAI is being adopted, the opportunities it presents, and the challenges it poses for teaching and learning. However, these reviews have largely conceptualized AI as an educational tool or educational problem. Comparatively little attention has been directed toward how the literature frames the broader educational purpose of preparing nurses for professional practice in health care systems where AI is already integrated into routine clinical care.

This distinction is important because educational discourse is not neutral. The ways in which emerging technologies are framed influence curriculum priorities, assessment practices, faculty development, and ultimately the capabilities that nursing programs seek to cultivate. When AI is positioned primarily as a threat to academic integrity, educational responses naturally focus on governance, surveillance, and assessment redesign. While these responses are appropriate and necessary, they risk narrowing the educational conversation by privileging academic compliance over the broader question of professional formation. Understanding how the literature conceptualizes the educational challenge posed by GenAI is therefore essential, as these conceptualizations shape what is valued, taught, and assessed within nursing education.

Professional identity formation provides a useful theoretical lens through which to examine this issue. Professional identity formation describes the developmental process through which learners progressively integrate the values, behaviors, knowledge, and responsibilities of the profession into their sense of self, ultimately learning to “think, act, and feel” like nurses [12,13]. In nursing, this process encompasses the development of clinical judgment, ethical reasoning, patient advocacy, relational care, accountability, and moral agency [14]. As AI becomes increasingly embedded within health care delivery, the contexts in which professional identity develops are also changing. Consequently, understanding how nursing education conceptualizes AI extends beyond questions of technology adoption to encompass how future nurses are prepared to exercise judgment, maintain professional responsibility, and provide person-centered care alongside increasingly sophisticated algorithmic systems.

Against this background, this review adopts a different perspective from previous syntheses. Rather than asking how GenAI is being used in nursing education or whether its use should be encouraged or restricted, this review examines how the nursing education literature conceptualizes the educational challenge itself. Specifically, it explores whether contemporary discourse remains predominantly centered on academic integrity and educational governance or whether it also addresses professional formation for algorithmically mediated clinical practice.

The review was guided by the following research question: how is GenAI conceptualized within the nursing education literature and to what extent does the current discourse address professional formation?

Professional identity formation therefore served as the conceptual lens underpinning the review, informing both the analysis of the literature and the interpretation of how nursing education currently positions students as future professionals within increasingly AI-enabled health care environments.

## Methods

### Design

This study used an integrative review methodology guided by Whittemore and Knafl’s [15] framework, which supports the systematic synthesis of diverse forms of evidence, including empirical research, reviews, theoretical papers, commentaries, editorials, and professional guidance documents. This approach was selected because scholarship related to GenAI in nursing education remains methodologically heterogeneous and conceptually emergent, making conventional systematic review approaches that focus exclusively on empirical effectiveness studies insufficient to address the research question.

Unlike reviews designed to evaluate intervention effectiveness, this review sought to examine how GenAI is conceptualized across contemporary nursing education literature. Accordingly, the review combined systematic literature identification with qualitative interpretive synthesis to explore the dominant narratives, assumptions, and conceptual framings underpinning current scholarship.

### Conceptual Orientation

Professional identity formation served as the conceptual lens underpinning the review. Rather than cataloging educational applications of GenAI, this review examined how nursing education scholarship constructs the educational challenges associated with AI and the extent to which these constructions address preparation for algorithmically mediated clinical practice.

This orientation recognizes that literature not only reports evidence but also shapes professional priorities by privileging particular educational concerns while rendering others less visible. Consequently, the review adopted a discourse-oriented approach, seeking to identify both dominant conceptual framings and important conceptual absences within the literature.

### Search Strategy

A systematic literature search was undertaken to identify publications examining GenAI within nursing education. Searches were conducted across PubMed, Google Scholar, and SciSpace (Typeset), capturing literature published between January 1, 2022, and February 28, 2026. The search period commenced immediately prior to the public release of ChatGPT to capture the emergence of contemporary GenAI scholarship.

The database selection reflected complementary strengths. PubMed was selected because it comprehensively indexes biomedical and health professions literature. Google Scholar provides broad scholarly coverage across disciplines, while SciSpace facilitates the retrieval of recently published and emerging AI-related scholarship in this rapidly evolving field.

The search terms combined controlled vocabulary (where available) and keywords relating to GenAI, nursing education, and professional formation. Boolean operators were used to combine concepts, with database-specific adaptations applied as required. The complete search strategies for all databases are presented in Multimedia Appendix 1.

Following database searches, the reference lists of included reviews and key papers were manually examined to identify additional eligible studies not retrieved through electronic searches. Textbox 1 summarizes the eligibility criteria.

Textbox 1. **Inclusion and exclusion criteria.**

**Inclusion criteria**:

- Papers addressing generative AI in nursing education contexts

- Published or preprinted between 2022 and 2026

- Focus on undergraduate or graduate nursing education

- Empirical studies, theoretical papers, position statements, or reviews

**Exclusion criteria**:

- Papers focusing solely on nongenerative AI (eg, predictive analytics and diagnostic imaging AI)

- Papers addressing AI in clinical practice without educational implications

- Conference abstracts without full text

- Duplicate publications

### Study Selection

All search results were imported into a reference management program, and duplicate records were removed prior to screening.

Study selection followed the PRISMA (Preferred Reporting Items for Systematic Reviews and Meta-Analyses) 2020 framework (Checklist 1). Titles and abstracts were independently screened by 2 reviewers against the predefined eligibility criteria. Potentially eligible studies subsequently underwent full-text assessment.

Disagreements at either stage were resolved through discussion until consensus was reached. Where uncertainty remained regarding eligibility, studies were retained for full-text review to maximize sensitivity during screening.

The study selection process is presented in the PRISMA flow diagram in the *Results* section.

### Quality Appraisal

Methodological quality was assessed for all empirical studies using the Mixed Methods Appraisal Tool (MMAT), which allows consistent appraisal across qualitative, quantitative, and mixed methods research designs.

Because this review also included reviews, theoretical papers, editorials, and professional commentaries, these publications were appraised according to their conceptual relevance, clarity of argument, and contribution to the understanding of GenAI within nursing education rather than being evaluated with empirical quality criteria.

Consistent with integrative review methodology, studies were not excluded on the basis of methodological quality. Instead, quality appraisal informed the interpretation of the findings by providing greater weight to higher-quality empirical evidence while recognizing the important conceptual contributions of theoretical literature.

### Data Extraction

A structured data extraction framework was developed prior to the analysis and was applied consistently across all included publications.

For each study, the following information was extracted: bibliographic details, year of publication, country of origin, publication type, study design, educational context, primary focus of the AI application, principal findings, and relevance to professional formation.

To support the review’s conceptual focus, each paper was additionally coded for the presence or absence of 7 predefined professional formation constructs derived from the literature. Coding was based on explicit discussion of each construct within the publication. Where constructs were not directly named, studies were coded as present only when the underlying concept was clearly described or operationalized in relation to nursing education. The predefined professional formation constructs comprised professional identity formation, ethical reasoning, moral agency, clinical judgment, digital professionalism, algorithmic bias and power awareness, and preparation for algorithmically mediated clinical practice.

Studies were further coded according to publication characteristics, dominant AI framing, educational focus, and the presence of the predefined professional formation constructs. Descriptive coding captured publication characteristics and educational context, while conceptual coding identified how GenAI was framed within the literature and whether professional formation constructs were explicitly or implicitly represented. Coding categories were initially informed deductively by the professional identity formation literature and subsequently refined through iterative familiarization and engagement with the corpus.

The complete extraction framework is presented in Multimedia Appendix 2.

### Data Analysis

Analysis occurred in 2 complementary phases.

#### Phase 1: Corpus-Level Descriptive Mapping

The first phase involved corpus-level descriptive mapping across all included publications.

Descriptive analyses quantified publication trends, dominant conceptualizations of GenAI, and the frequency with which professional formation constructs appeared across the literature. These analyses provided an overview of the prevailing discourse within contemporary nursing education scholarship.

#### Phase 2: Interpretive Synthesis

The second phase used interpretive synthesis to examine how the identified patterns collectively constructed the discourse surrounding GenAI within nursing education.

Rather than synthesizing findings study-by-study, the interpretive analysis examined relationships across the corpus to identify dominant narratives, underlying assumptions, conceptual tensions, and important omissions. Constant comparison was used throughout the analysis, with emerging interpretations repeatedly tested against the complete dataset to ensure that they adequately represented the breadth of the included literature.

Professional identity formation served as the interpretive framework through which dominant conceptualizations were examined, enabling the identification of both explicit discussions and notable silences relating to identity development, moral agency, clinical judgment, and preparation for algorithmically mediated practice.

The integration of corpus-level descriptive mapping with interpretive synthesis enabled the review to move beyond describing what has been published toward understanding how the field currently conceptualizes the educational implications of GenAI.

To enhance analytical rigor, all included publications were independently coded by 2 reviewers using the predefined coding framework. Coding decisions were subsequently compared, with any discrepancies discussed and resolved through consensus. Throughout the analytical process, category definitions and interpretations were iteratively refined to ensure consistency across the corpus while remaining grounded in the included literature. Given the interpretive nature of this integrative review, formal intercoder reliability statistics were not calculated. Instead, analytical trustworthiness was enhanced through independent coding, iterative discussion, and consensus-based refinement of the coding framework, consistent with established integrative review methodology.

## Results

### Study Characteristics

#### Overview of the Included Literature

The study selection process is presented in Figure 1. Database searches identified 456 records, of which 87 duplicates were removed. Following title and abstract screening, 250 records were excluded, leaving 119 publications that met the eligibility criteria and were included in the review.

**Figure 1.**
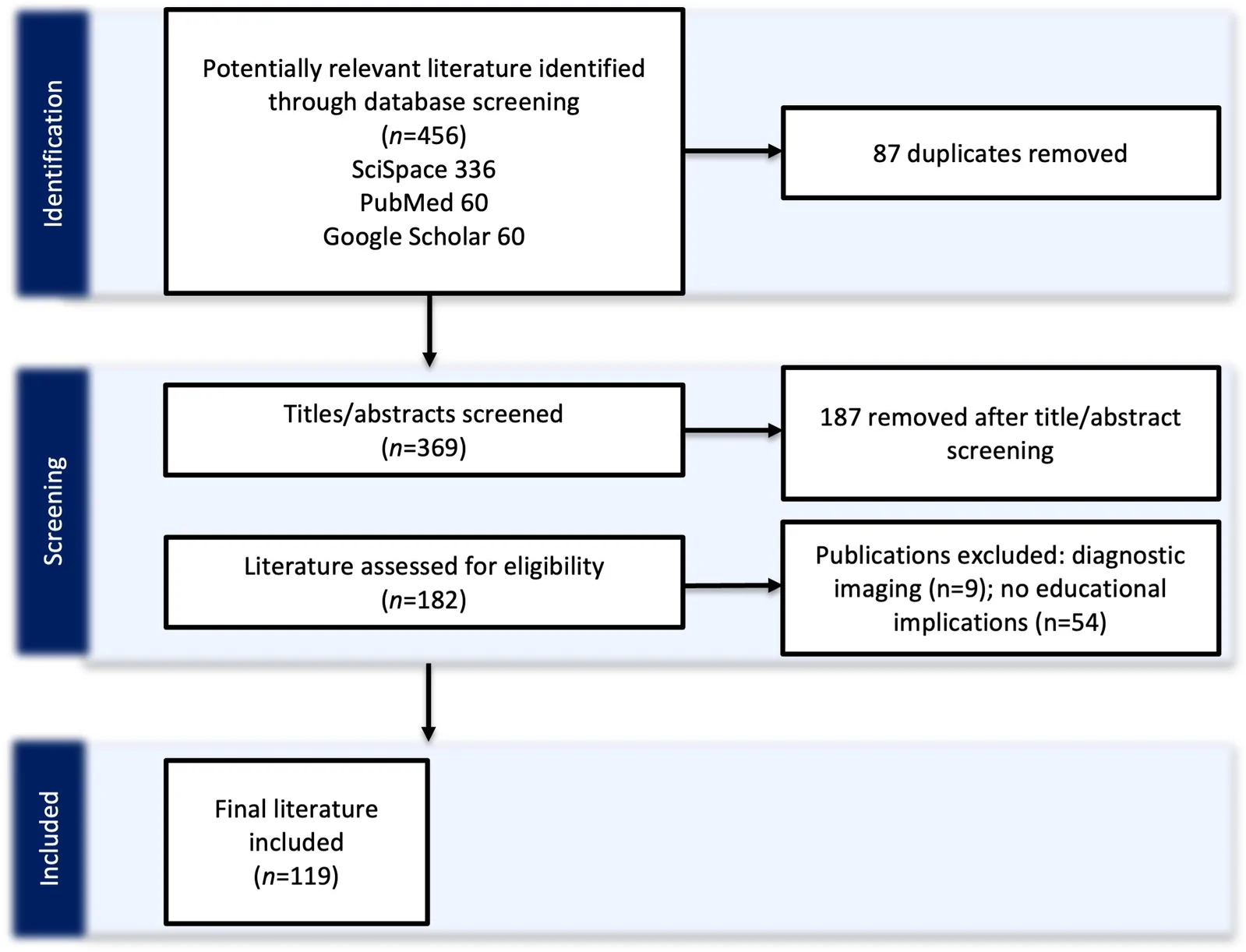
PRISMA (Preferred Reporting Items for Systematic Reviews and Meta-Analyses) flow diagram.

Of the 119 publications, 64 (53.8%) were empirical studies, 31 (26.1%) were scoping or systematic reviews, 13 (10.9%) were position papers or commentaries, and 11 (9.2%) were theoretical or framework papers. Publications originated from multiple geographical regions, with the strongest representation from the United States, China, Australia, and Europe. Publication volume increased rapidly over the review period, rising from 8 publications during 2022‐2023 to 43 in 2024 and 68 in 2025‐2026, reflecting the rapid expansion of scholarly interest following the public release of GenAI technologies (Table 1).

**Table 1.** Characteristics of included studies (n=119).

Characteristic	Values, n (%)
Empirical studies	64 (53.8)
Scoping/systematic reviews	31 (26.1)
Position papers/commentaries	13 (10.9)
Theoretical/framework papers	11 (9.2)
Publications by year	
2022‐2023	8 (6.7)
2024	43 (36.1)
2025‐2026	68 (57.1)

Rather than presenting isolated themes, the findings are organized around the dominant ways in which GenAI is conceptualized within the nursing education literature. Corpus-level mapping identified recurring conceptual framings across the included publications, while interpretive synthesis examined how these framings positioned students, educators, and professional preparation for algorithmically mediated practice. Figure 2 presents the resulting conceptual model that informed the thematic synthesis.

The included literature comprised empirical studies (n=64), scoping and systematic reviews (n=31), position papers and commentaries (n=13), and theoretical or framework papers (n=11). Geographic representation was global, with significant contributions from the United States, China, Australia, and Europe. Publication frequency increased sharply over time, with 8 papers in 2022‐2023, 43 in 2024, and 68 in 2025‐2026, reflecting rapid scholarly response following the public release of GenAI technologies.

Figure 2 presents a conceptual model derived from the integration of corpus-level mapping and interpretive synthesis. The model illustrates a governance-pedagogy cycle anchored in integrity discourse, wherein AI is framed primarily as an integrity threat or a pedagogical instrument, generating governance and literacy-based responses. Professional formation in algorithmically mediated practice remains comparatively underintegrated within this cycle. The themes that follow elaborate on each component of this discourse cycle.

**Figure 2.**
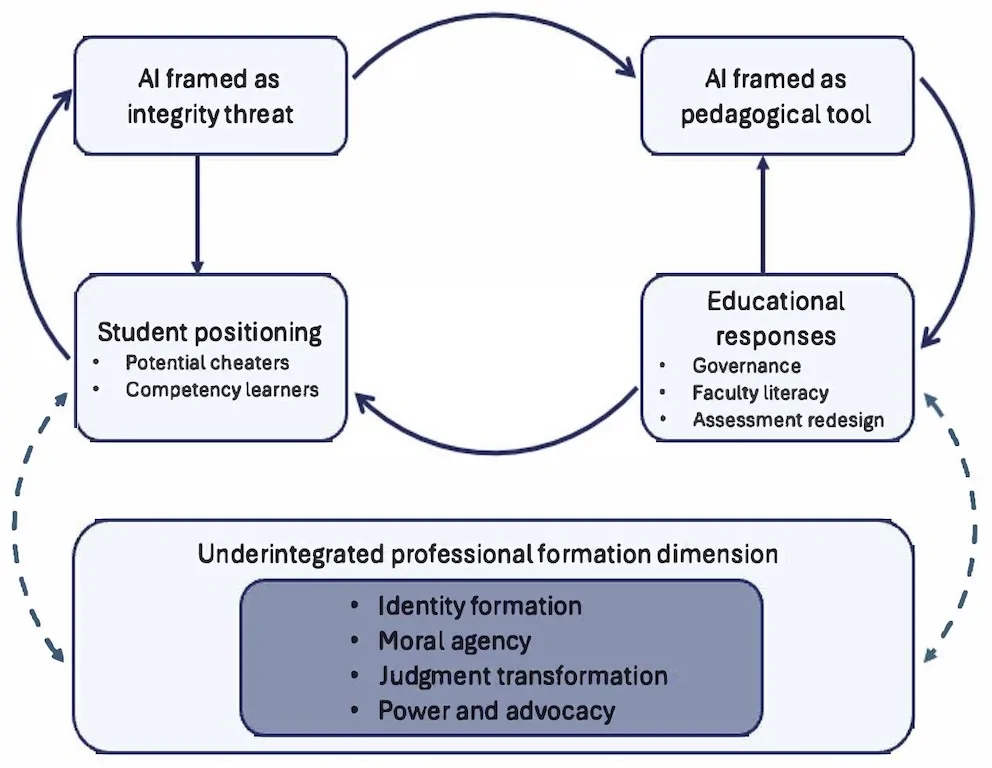
Conceptual model of generative AI discourse in nursing education. Synthesis revealed a dominant tool-oriented discourse cycle and comparatively limited integration of professional formation in algorithmically mediated practice.

#### Theme 1: AI as a Writing Tool and an Integrity Threat

The dominant discourse positioned GenAI primarily as a content-generation tool with implications for academic integrity, assessment validity, and authorship authenticity. Across the corpus, AI was most frequently discussed in relation to plagiarism, inappropriate assistance with assessments, and concerns regarding the integrity of student work, rather than its influence on professional preparation or future clinical practice.

Shorey et al [3] described ChatGPT as raising concerns regarding plagiarism, academic dishonesty, and the authenticity of student work, while Siah et al [4] similarly framed authenticity and academic integrity as the central educational challenges requiring investigation. Across the literature, 3 recurring subthemes characterized this integrity-focused discourse.

The first concerned assessment validity. Many publications questioned whether AI-generated work undermined assessment as a measure of student learning and clinical competence, often equating undisclosed AI use with plagiarism or other forms of academic misconduct [3-5]. The second focused on detection and governance, with considerable attention devoted to AI detection software, institutional policies, disclosure requirements, and faculty responsibilities for regulating AI use [5,16]. Finally, numerous publications advocated assessment redesign, recommending oral examinations, in-person assessments, authentic assessment tasks, and reflective activities that were perceived to be less vulnerable to AI-assisted completion [3,4,17].

Collectively, these studies positioned academic integrity as the principal educational problem associated with GenAI. Although this framing acknowledged legitimate concerns regarding assessment authenticity, professional formation received comparatively limited attention within this dominant discourse.

#### Theme 2: AI as a Pedagogical Tool

Alongside the dominant integrity discourse, a smaller but growing body of literature conceptualized GenAI as a pedagogical resource capable of enhancing learning rather than threatening assessment integrity. These publications shifted attention from regulation and surveillance toward constructive educational integration.

Several pedagogical applications recurred throughout the corpus. AI was described as supporting clinical reasoning through the generation of patient scenarios, care plans, and differential diagnoses that students were required to critically evaluate [18-20]. Other studies emphasized personalized learning, describing AI systems that provided tailored feedback, adaptive learning opportunities, and conversational support for evidence-based practice [21,22]. Additional publications highlighted opportunities to improve accessibility, particularly for students with language barriers or diverse learning needs, while others positioned AI literacy itself as an emerging professional capability required for contemporary nursing practice [1,21,22].

Despite representing a more constructive framing than the integrity-focused literature, this discourse remained predominantly educational in orientation. AI was generally conceptualized as a teaching and learning resource rather than as a feature of contemporary clinical environments requiring explicit preparation for professional practice.

#### Theme 3: The Missing Discourse on Professional Formation in Algorithmic Practice

While integrity and pedagogy dominated the literature, a different pattern also emerged. Across the corpus, comparatively little attention was directed toward how AI might reshape the formation of nurses as professionals practicing within algorithmically mediated health care systems. Seven dimensions of professional formation were examined during the analysis, including identity formation, ethical reasoning, moral agency, clinical judgment, digital professionalism, awareness of algorithmic power, and recognition of AI within contemporary clinical practice.

### Identity Formation, Moral Agency, and Clinical Judgment

Professional identity formation was explicitly addressed in only 12 of the 119 (10%) included publications. When identity was discussed, students were typically positioned as learners developing AI-related competencies rather than professionals negotiating evolving clinical roles and responsibilities. References to professional identity were commonly embedded within discussions of digital professionalism, focusing on appropriate AI use and disclosure practices rather than the broader developmental process through which nurses learn to think, act, and feel as members of the profession.

Moral agency was even less visible. Only 6 (5%) publications explicitly discussed nurses’ responsibility to question, challenge, or override algorithmic recommendations when patient welfare required it. Although professional accountability was occasionally acknowledged, few studies described educational strategies for cultivating the moral courage needed to exercise independent clinical judgment within AI-supported practice environments.

Clinical judgment appeared more frequently, with 38 (31.9%) publications referring to critical thinking or decision-making. However, these discussions largely focused on evaluating AI-generated outputs rather than examining how algorithmic systems may reshape clinical reasoning itself. AI was commonly positioned as a learning scaffold supporting judgment, rather than as a technology transforming the contexts in which professional judgment is exercised.

### Ethical Awareness, Power, and the Algorithmic Present

#### AI Ethics and Clinical Preparedness

Ethical issues were identified in approximately 38% of the corpus and were most commonly related to algorithmic bias, privacy, transparency, and the reliability of AI-generated information [9,11,23,24]. These discussions generally emphasized vigilance, verification, and technical oversight, with relatively few publications describing structured educational approaches to developing ethical reasoning in AI-supported clinical practice.

Similarly, approximately one-third of publications acknowledged algorithmic bias, inequity, or opacity, yet few translated these concerns into curricular approaches that prepared students to recognize, challenge, or respond to inequitable algorithmic decision-making. Student concerns regarding AI bias were evident in empirical studies, although participants frequently reported limited preparation for managing these issues in practice.

A further finding was the limited recognition that AI is already embedded within many contemporary health care systems. Across much of the literature, AI was discussed as an emerging or future technology despite its established role in early warning systems, clinical decision support, risk stratification, and documentation technologies. Taken together, these findings suggest that while ethical and technological issues receive growing attention, professional formation within algorithmically mediated health care remains comparatively underdeveloped.

#### Theme 4: Student Identity Construction From Threat to Opportunity

The analysis identified 4 dominant ways nursing students were positioned within the literature. First, students were frequently portrayed as potential academic integrity risks, requiring surveillance and regulation to prevent inappropriate AI use. Second, students were described as digitally capable but requiring guidance, reinforcing the faculty’s responsibility for establishing appropriate boundaries and institutional expectations. Third, students were positioned as future professionals developing AI-related competencies, although competency development was generally framed in technical rather than professional terms. Finally, empirical studies portrayed students as pragmatic users seeking to integrate AI into their learning while navigating uncertainty regarding institutional expectations.

Across these constructions, relatively little attention was directed toward students as professionals-in-formation preparing to practice alongside algorithmic technologies. Consequently, the literature rarely articulated how future nurses might develop professional judgment, moral agency, and relational practice within AI-enabled health care environments.

#### Theme 5: Proposed Educational Responses Involving Governance, Literacy, and Pedagogy

Three broad categories of educational response emerged across the literature: governance and policy development, faculty AI literacy, and pedagogical innovation. These responses largely reflected the dominant conceptual framings identified throughout the corpus.

Governance-focused publications advocated institutional policies, disclosure requirements, academic integrity training, and faculty guidance to regulate AI use within assessment [5,16,25]. Faculty development initiatives primarily focused on improving educators’ technological knowledge, assessment design, and confidence in integrating AI into teaching [1,10,26]. Pedagogical innovations included AI-generated patient scenarios, structured evaluation of AI outputs, personalized feedback systems, and collaborative learning approaches in which students interacted directly with AI technologies [18-22,27,28].

Although these responses demonstrated increasing educational sophistication, they generally remained situated within existing curricular structures. Few publications proposed sustained curriculum transformation centered on professional identity formation, clinical judgment, or moral agency within algorithmically mediated practice environments.

## Discussion

### Principal Findings

The central finding of this review is a significant misalignment between how GenAI is framed within the nursing education literature and the realities of contemporary clinical practice. Among the 119 publications, the discourse is dominated by concerns about academic integrity (n=100, 84%), whereas comparatively little attention is given to professional identity formation (n=12, 10%) or moral agency (n=6, 5%). This imbalance suggests that nursing education is responding to GenAI primarily as an assessment problem rather than as a transformation of professional practice environments. Interpreting these findings through the lens of professional identity formation highlights how current discourse emphasizes governance and competency while underengaging with the developmental processes through which nurses learn to exercise judgment, agency, and relational care in technologically mediated practice environments.

Across the corpus, GenAI is most frequently conceptualized as a writing tool that threatens authorship authenticity and assessment validity [3-5]. This framing has generated extensive discussion about detection tools, policy frameworks, and assessment redesign [3,4,16]. While these concerns are legitimate, the prominence of this discourse risks positioning students primarily as potential rule-breakers rather than as emerging professionals who must develop judgment and ethical agency within algorithmically mediated health care systems.

Together, these findings identify an algorithmic formation gap in nursing education discourse. While the literature actively debates whether students should use AI in academic work, comparatively little attention is directed toward how nurses learn to practice alongside algorithmic systems in contemporary health care.

### The Integrity Discourse: Necessary but Insufficient

The prominence of academic integrity discourse is not surprising. Academic integrity represents a familiar institutional challenge for universities, with established responses including plagiarism detection, honor codes, and assessment redesign [3,4]. Within this framing, GenAI becomes a new technological manifestation of a longstanding concern about academic misconduct.

However, this regulatory orientation may also reflect what can be understood as problem displacement. Integrity is a manageable challenge that universities can address through policy development, surveillance technologies, and compliance mechanisms [5]. By contrast, the deeper challenge posed by AI involves reconsidering how professional education prepares students for clinical practice environments increasingly shaped by algorithmic decision-support systems.

Empirical research suggests that students themselves often approach GenAI pragmatically. Rather than using AI primarily to circumvent learning, many students report using it as a study aid while expressing uncertainty regarding institutional expectations and professional boundaries [29-31]. An educational response focused primarily on prevention and detection may therefore overlook an important opportunity: developing the professional judgment required to navigate AI responsibly within clinical practice.

Integrity remains an essential foundation of professional practice. However, integrity discourse alone cannot adequately address the broader transformation of health care environments in which nurses must interpret, question, and sometimes override algorithmic recommendations.

### The Algorithmic Formation Gap

If professional education aims to develop individuals who “think, act, and feel” like members of the profession [12], then the integration of AI raises fundamental questions for professional formation in nursing.

Professional identity formation involves the integration of professional values, knowledge, and behaviors into an individual’s sense of self [12,13]. When clinical practice environments incorporate algorithmic decision-support systems, automated risk stratification tools, and AI-assisted documentation technologies, the context in which nurses develop professional identities is fundamentally altered.

This review suggests that nursing education discourse has not yet fully engaged with this transformation. While the literature acknowledges ethical and technological challenges associated with AI, sustained attention to how nurses develop professional identities within algorithmically mediated care environments remains limited.

It is important to acknowledge that nursing is not alone in responding to the educational implications of AI. Medical education has similarly focused on AI literacy, ethical considerations, governance, and the integration of AI into clinical reasoning curricula. More recently, however, medical education scholarship has begun to extend beyond technical competency by considering human-AI collaboration, professional responsibility, and professional identity formation as integral components of AI-enabled practice [6]. Likewise, broader work examining clinical decision-support systems has highlighted how algorithmic technologies may reshape clinicians’ autonomy, judgment, and professional identity [7]. These developments suggest that professional formation is becoming an increasingly recognized concern across the health professions. However, the present review indicates that this perspective remains comparatively underdeveloped within the nursing education literature, where discourse continues to be dominated by academic integrity, assessment validity, and technological competency. Rather than positioning nursing as uniquely deficient, the findings suggest that nursing is participating in a broader transition across health professions education while highlighting opportunities for the discipline to more explicitly integrate professional identity formation into AI-related curriculum development.

The implications of this formation gap can be understood across 3 interrelated dimensions: thinking, acting, and feeling as a professional nurse.

### Thinking: Clinical Judgment in Algorithmic Contexts

Clinical judgment is a foundational capability of professional nursing practice. Tanner’s model of clinical judgment emphasizes the integration of noticing, interpreting, responding, and reflecting in complex patient situations [32].

The literature reviewed here frequently emphasizes the importance of critical evaluation of AI-generated outputs [18,19]. These approaches are valuable in developing analytical reasoning skills. However, they primarily frame clinical judgment as a capacity applied to algorithmic recommendations rather than as a capability that may itself be reshaped by algorithmic practice environments.

Algorithmic decision-support systems can introduce risks such as automation bias, in which clinicians defer to automated recommendations even when conflicting evidence is present [33]. Similarly, excessive reliance on automated outputs may contribute to deskilling, whereby clinicians’ independent pattern recognition and intuition gradually diminish [34].

Preparing students for algorithmically mediated care therefore requires more than the ability to critique AI outputs. It requires developing the capacity to integrate algorithmic information into clinical reasoning while maintaining independent professional judgment.

### Acting: Moral Agency and Professional Courage

A second dimension of professional formation concerns moral agency as the ability to act on ethical conclusions even when doing so requires challenging institutional or technological authority.

Nursing has long emphasized patient advocacy as a central professional responsibility [35]. In algorithmic contexts, advocacy may involve questioning automated recommendations, identifying bias within predictive models, or prioritizing individualized patient needs over standardized algorithmic outputs.

Despite the ethical concerns identified across the literature, including bias, transparency, and privacy, few papers address how nurses develop the moral courage required to challenge algorithmic decision-support systems when patient welfare demands it [9,11,23,36]. Ethical awareness alone is insufficient if it does not translate into the capacity for moral action.

Professional formation in the age of AI must therefore include opportunities for students to practice exercising judgment and advocacy in situations where algorithmic recommendations conflict with clinical assessment.

### Feeling: Relational Care in Technologically Mediated Environments

A third dimension of professional identity formation concerns the relational and affective aspects of nursing practice. Nursing has historically defined itself through commitments to compassion, presence, and holistic patient care.

The integration of algorithmic technologies introduces new forms of technological mediation between nurses and patients. Clinical dashboards, automated alerts, and AI-assisted documentation systems may shift the focus of clinical attention from the bedside to the digital interface.

This shift raises important questions regarding how nurses sustain relational care and moral responsibility when clinical decision-making becomes increasingly mediated by technological systems. Professional formation must therefore address how nurses maintain human connection, empathy, and patient advocacy within technologically complex health care environments.

### Addressing the Algorithmic Present

A striking observation from this review is the limited acknowledgment that AI is already embedded within contemporary health care delivery. Across the literature, AI is frequently framed as an emerging or future technology rather than as an existing component of clinical infrastructure.

In reality, algorithmic systems already influence many aspects of clinical practice, including sepsis prediction models, early warning systems, risk stratification tools, and automated documentation support within electronic health records. These systems shape triage decisions, clinical workflows, and resource allocation across health care settings.

The relative absence of this “algorithmic present” within nursing education discourse may reflect disciplinary boundaries or technological invisibility, where background computational systems are not recognized as AI by educators and clinicians. However, preparing students for a technological future while underengaging with the algorithmic realities of contemporary practice risks widening the formation gap identified in this review.

Addressing this gap requires stronger partnerships between clinical environments and nursing education to ensure that curricula reflect the technological infrastructures already shaping health care delivery.

### Toward a Professional Formation Framework

Addressing the algorithmic formation gap requires reframing AI integration as a professional formation challenge rather than a technological or regulatory issue.

This reframing shifts the focus from detecting inappropriate AI use toward preparing nurses to practice ethically and effectively within algorithmically mediated health care systems.

Table 2 outlines 5 conceptual shifts that may support this reframing. These shifts involve moving from integrity-focused discourse to curricular approaches that emphasize identity formation, moral agency, and professional judgment in technological contexts.

Examples of such approaches may include simulation scenarios involving algorithmic failures, structured reflection on professional identity in technologically mediated practice, and case-based ethical discussions involving AI-supported clinical decision-making.

Rather than treating AI as a discrete technological topic, nursing education may need to integrate AI considerations longitudinally across curricula, linking technological engagement with professional values, clinical reasoning, and patient advocacy.

**Table 2.** Proposed conceptual shifts for AI in nursing education.

Integrity/tool discourse	Professional formation framework
AI as an integrity threat	AI as a professional practice context
Tool competency and literacy	Identity formation (think/act/feel)
Ethical awareness/principles	Moral agency and professional resistance
Critical evaluation of output	Transformation of clinical judgment
Future technological preparation	Engagement with current algorithmic reality

### Implications for Nurse Educators

For nurse educators, the findings of this review suggest that GenAI should not be addressed solely through academic integrity policies or isolated technological skills training. Instead, AI must be integrated into curricula as part of professional formation. This includes creating learning experiences in which students critically engage with algorithmic decision-support systems, reflect on their evolving professional identities in technologically mediated environments, and practice exercising clinical judgment when AI recommendations conflict with patient needs. By situating AI within professional formation rather than academic compliance, educators can better prepare graduates for contemporary health care systems in which algorithmic tools increasingly shape clinical practice.

### Implications for Nursing Education

The question facing nursing education is no longer whether students should use AI in their coursework. The more pressing question is how nurses will exercise judgment, advocacy, and relational care within health care systems increasingly shaped by algorithmic decision-making.

Preparing students for these environments requires moving beyond governance and literacy toward professional formation. This includes creating opportunities for students to engage critically with AI systems, reflect on their evolving professional identities, and practice exercising clinical judgment in technologically complex care environments.

By reframing AI integration as a professional formation challenge, nursing education can ensure that graduates possess not only the technical literacy to interact with algorithmic systems but also the ethical judgment, moral courage, and relational capacity required to advocate for patients within them.

### Limitations

This review has several limitations. First, the rapid evolution of GenAI means that relevant literature published or in press after the search period may not have been captured. Given the pace of scholarly and technological developments in this field, findings should be interpreted as a time-sensitive snapshot of the discourse between 2022 and early 2026. Second, the search was limited to English-language publications and 3 databases (PubMed, Google Scholar, and SciSpace). Although these sources captured a broad and diverse corpus, relevant studies indexed elsewhere or published in other languages may have been missed. Third, the integrative review design intentionally included heterogeneous evidence types, such as empirical studies, reviews, theoretical papers, and commentaries. While this breadth enabled conceptual mapping of the field, the inclusion of nonempirical literature may limit direct comparability across studies and may amplify emerging opinion-based discourse. Fourth, the analysis relied on explicit reporting within published papers. The absence of discussion regarding professional identity formation, moral agency, or recognition of clinical AI infrastructure in the literature does not necessarily indicate the absence of these considerations in educational practice; rather, it reflects their limited articulation within the published discourse. Finally, the interpretive synthesis involved researcher judgment in coding and thematic interpretation. To enhance rigor, independent coding and reviewer discussion were used to refine categories and resolve disagreements.

### Conclusions

This integrative review identifies a fundamental algorithmic formation gap in nursing education discourse. The literature predominantly frames AI as a content-generation tool and academic integrity threat, with extensive attention to detection, prevention, and policy frameworks. While these concerns are legitimate, the disproportionate focus on integrity may obscure a more critical challenge: preparing nursing students for professional practice in algorithmically mediated health care environments.

The literature’s limited engagement with professional identity formation, moral agency, clinical judgment transformation, and the reality of algorithmic clinical practice represents a critical gap. Nursing education cannot adequately prepare students for contemporary practice if it treats AI primarily as a writing tool to be regulated rather than as a defining context for professional formation.

The absence of professional formation discourse is not merely a gap to be filled with additional content or competencies. It represents an argument for fundamental curricular transformation. If AI already mediates triage, diagnostics, care pathways, and clinical decision-making in practice settings and the evidence from health care delivery suggests it does, then nursing education must explicitly address how students develop professional identity, clinical judgment, and moral agency within these algorithmic contexts.

This transformation requires moving beyond the question “how do we prevent students from cheating with AI?” to the more fundamental question “how do we prepare students to think, act, and feel like nurses in algorithmically mediated care environments?” It requires recognizing that professional formation in the age of AI is not about acquiring discrete competencies but about developing the capacity to exercise clinical judgment, moral agency, and relational practice alongside algorithmic systems.

The nursing profession has long defined itself through commitments to patient advocacy, holistic care, clinical judgment, and moral courage. As algorithms increasingly mediate health care delivery, these professional commitments are not obsolete; they are more essential than ever. However, they require explicit, sustained, and curricular attention to how they are enacted in algorithmic contexts.

Nursing education stands at a critical juncture. Continuing to treat AI primarily as an integrity threat leaves graduates unprepared for the algorithmic reality of modern health care. By reframing AI integration as a professional formation challenge, nursing education can ensure that the next generation of nurses possesses not only the literacy to use AI but also the judgment and agency to advocate for patients within it.

## Supplementary material

10.2196/99284 Multimedia Appendix 1 Search strategy.

10.2196/99284 Multimedia Appendix 2 Data extraction table.

10.2196/99284 Checklist 1 PRISMA 2020 checklist.

## Abbreviations

GenAI: generative AI
MMAT: Mixed Methods Appraisal Tool
PRISMA: Preferred Reporting Items for Systematic Reviews and Meta-Analyses

## References

[1] Gonzalez L (2024). Nursing education in the era of ChatGPT: implications and opportunities. Online J Issues Nurs.

[2] Topaz M, Peltonen LM, Michalowski M (2025). The ChatGPT effect: nursing education and generative artificial intelligence. J Nurs Educ.

[3] Shorey S, Mattar C, Pereira TLB, Choolani M (2024). A scoping review of ChatGPT’s role in healthcare education and research. Nurse Educ Today.

[4] Siah CJR, Lim JV, Lyu MM, Levett-Jones T, Mattar I (2026). Authenticity and academic integrity in generative artificial intelligence (GenAI) use among undergraduate nursing students. J Prof Nurs.

[5] Labrague LJ (2026). A five-tier framework for guiding responsible AI use in nursing students’ coursework: a faculty guide. Teach Learn Nurs.

[6] Fu C, Li J, Fan H (2026). AI literacy in undergraduate medical education: a competency-based interpretive framework for curriculum and assessment. Front Med (Lausanne).

[7] Ackerhans S, Huynh T, Kaiser C, Schultz C (2024). Exploring the role of professional identity in the implementation of clinical decision support systems-a narrative review. Implement Sci.

[8] Amankwaa I, Ekpor E, Cudjoe D (2025). Patterns, advances, and gaps in using ChatGPT and similar technologies in nursing education: a PAGER scoping review. Nurse Educ Today.

[9] Hardie P, Darley A, Derwin R (2026). Applications, attitudes and ethical considerations of generative artificial intelligence (Gen AI) in nursing education: a scoping review. BMC Nurs.

[10] Hinsche L, Hasseler M, Tischendorf T, Schaal T (2025). Rapid review on GenAI in nursing education. Front Health Serv.

[11] Luque Loor AH, Cedeño Orejuela JA, Melis Sosa A (2025). Implications of generative artificial intelligence on ethics in nursing education: state of the art review [Article in Spanish]. Rev Esp Edu Med.

[12] Cruess RL, Cruess SR, Boudreau JD, Snell L, Steinert Y (2014). Reframing medical education to support professional identity formation. Acad Med.

[13] Cruess RL, Cruess SR, Steinert Y (2016). Amending Miller’s pyramid to include professional identity formation. Acad Med.

[14] Browne CA, Fetherston CM (2018). How do we facilitate international clinical placements for nursing students: a cross-sectional exploration of the structure, aims and objectives of placements. Nurse Educ Today.

[15] Whittemore R, Knafl K (2005). The integrative review: updated methodology. J Adv Nurs.

[16] Chen SL, Lee LL (2024). Strengths, weaknesses, opportunities, and threats analysis of integrating ChatGPT into nursing education. Hu Li Za Zhi.

[17] Stratton-Maher D, Lindsay J, Jacka L (2024). eBook of Shortened Peer Reviewed Papers.

[18] Simms RC (2024). Work with ChatGPT, not against: 3 teaching strategies that harness the power of artificial intelligence. Nurse Educ.

[19] Fung TCJ, Chan SL, Lam CFM (2025). Effects of generative artificial intelligence (GenAI) patient simulation on perceived clinical competency among global nursing undergraduates: a cross-over randomised controlled trial. BMC Nurs.

[20] Hwang BY, Yu M (2026). From film to simulation: mixed-methods study of learner-driven artificial intelligence-generated ethical dilemma scenarios and role-play to enhance ethical competence in nursing students. Nurse Educ Pract.

[21] Go J, Lee Y (2026). The effectiveness of AI-based conversational agents in nursing education: a systematic review. Nurse Educ Pract.

[22] Yang S, Shi M, Qian Y (2026). A large language model-powered reflective AI agent for evidence-based nursing education: design and evaluation. Nurse Educ Pract.

[23] Yuan Y, Fu J, Leng L (2025). The strengths, weaknesses, opportunities, and threats of generative artificial intelligence: a qualitative study of undergraduate nursing students. Front Public Health.

[24] Rodrigues D, Cruz-Correia R (2024). Large language models in nursing education: state-of-the-art. Stud Health Technol Inform.

[25] Choi MJ, Seo MH, Kim J, Kim S, Jeong SH (2025). Research trends in generative artificial intelligence in nursing: a scoping review. J Korean Acad Nurs.

[26] Gerdes M, Bayne A, Henry K (2025). Emerging artificial intelligence-based pedagogies in didactic nursing education: a scoping review. Nurse Educ.

[27] Stratton-Maher D, Kelly J (2026). Learning with AI, not from AI: ethical and pedagogical implications of ChatGPT in undergraduate education. Nurse Educ Today.

[28] Vanderlaan J, Nicholas L, Leland N (2025). Practical intelligence: generative AI toolkit for nurse education. https://oasis.library.unlv.edu/nursing_fac_articles/424.

[29] Sajja R, Sermet Y, Demir I, Chi NC (2025). Evaluating artificial intelligence assisted nursing education: student perceptions, ethical concerns, and pedagogical implications. Teach Learn Nurs.

[30] Blomquist J, Alderden J, Llewellyn S, Haynes M, Dworak E (2025). Student and faculty perceptions and use of generative artificial intelligence in nursing education. J Nurs Educ.

[31] Summers A, Haddad ME, Prichard R, Clarke KA, Lee J, Oprescu F (2024). Navigating challenges and opportunities: nursing student’s views on generative AI in higher education. Nurse Educ Pract.

[32] Tanner CA (2006). Thinking like a nurse: a research-based model of clinical judgment in nursing. J Nurs Educ.

[33] Goddard K, Roudsari A, Wyatt JC (2012). Automation bias: a systematic review of frequency, effect mediators, and mitigators. J Am Med Inform Assoc.

[34] Parasuraman R, Manzey DH (2010). Complacency and bias in human use of automation: an attentional integration. Hum Factors.

[35] Lachman VD (2007). Moral courage in action: case studies. Medsurg Nurs.

[36] De Gagne JC, Hwang H, Jung D (2024). Cyberethics in nursing education: ethical implications of artificial intelligence. Nurs Ethics.

