# Supplementary material for: From Academic Integrity to Professional Formation in Generative AI–Supported Nursing Education: Integrative Review
Source: JMIR Nurs. 2026 Oct 30;9:e99284. doi: 10.2196/99284 (PMC13722097; doi:10.2196/99284)
Supplement: Supplementary file 2 [file nursing-v9-e99284-s001.docx]

**Multimedia Appendix 1**

**Search Strategy Details**

**PubMed Search String**

("generative AI"[Title/Abstract] OR "ChatGPT"[Title/Abstract] OR "large language model"[Title/Abstract] OR "artificial intelligence"[Title/Abstract] OR "GenAI"[Title/Abstract] OR "GPT-4"[Title/Abstract] OR "conversational AI"[Title/Abstract])
AND
("nursing education"[Title/Abstract] OR "nursing student"[Title/Abstract] OR "nursing curriculum"[Title/Abstract] OR "nursing pedagogy"[Title/Abstract] OR "undergraduate nursing"[Title/Abstract])
AND
("professional formation"[Title/Abstract] OR "professional identity"[Title/Abstract] OR "clinical judgment"[Title/Abstract] OR "academic integrity"[Title/Abstract] OR "clinical practice"[Title/Abstract] OR "algorithmic practice"[Title/Abstract] OR "student identity"[Title/Abstract])
Filters: Publication date from 2022/01/01 to 2026/02/28

**Google Scholar Search String**

("generative AI" OR "ChatGPT" OR "large language model" OR "artificial intelligence" OR "GenAI") AND ("nursing education" OR "nursing student" OR "nursing curriculum") AND ("professional formation" OR "professional identity" OR "clinical judgment" OR "academic integrity") 2022-2026

**SciSpace Search String**

Similar structure adapted to SciSpace interface with date range 2022-2026
