# Supplementary material for: From Academic Integrity to Professional Formation in Generative AI–Supported Nursing Education: Integrative Review
Source: JMIR Nurs. 2026 Oct 30;9:e99284. doi: 10.2196/99284 (PMC13722097; doi:10.2196/99284)
Supplement: Supplementary file 3 [file nursing-v9-e99284-s002.docx]

**Multimedia Appendix 2**

# Data Extraction Table: AI in Nursing Education (119 Papers)

| No. | Author(s) | Year | Title | Study Type | AI Focus | Formation Elements | Key Findings |
| --- | --- | --- | --- | --- | --- | --- | --- |
| 1. | Shefaly Shorey et al. | 2024 | A scoping review of ChatGPT’s role in healthcare education and research. | scoping review | AI in simulation/education. Specific AI technologies mentioned: ChatGPT. | * professional identity formation: Not available in metadata. * ethical reasoning: Present, indicated by “legitimate concerns related to the potential misuse of ChatGPT”, “confidentiality and security risks”, “controversial practice of crediting it as a co-author”, and “urgency of establishing timely guidelines and regulations”. * moral agency: Present, implied by “responsible and safe implementation of ChatGPT’s capabilities” and “urgency of establishing timely guidelines and regulations”. * clinical judgment in algorithmic contexts: Limited presence, mentioned in the context of “limited understanding of specialized healthcare knowledge” by ChatGPT, suggesting human judgment is still critical. * digital professionalism: Present, indicated by “confidentiality and security risks”, “data protection measures should be prioritized”, and “transparent reporting becomes crucial when integrating ChatGPT into academic writing”. * power/bias awareness: Not explicitly mentioned. * recognition that AI mediates clinical decisions: Limited presence, implied by “limited understanding of specialized healthcare knowledge” and the focus on its role in education and research rather than direct clinical decision mediation. * preparation for algorithmic practice: Present, indicated by “adaptation of higher education curricula to incorporate ChatGPT’s potential” and “educators’ need to familiarize themselves with the technology to improve their literacy and teaching approaches”. | This review highlights significant concerns regarding ChatGPT’s potential misuse, its limited specialized healthcare knowledge, and its impact on teaching methods and assessments, alongside confidentiality and security risks. It emphasizes the urgent need for timely guidelines, regulations, and active stakeholder engagement to ensure responsible and safe implementation. Recommendations include using cross-verification, adapting higher education curricula, familiarizing educators with the technology, and prioritizing data protection measures. |
| 2. | Hinsche et al. | 2025 | Rapid review on GenAI in nursing education. | Rapid review | The primary AI focus is on multiple AI applications in nursing education, specifically generative AI (represented by ChatGPT) for personalized learning, simulation training, and teaching process support. It also addresses AI ethics/bias, digital competence, and potential risks like distortion of knowledge and educational inequalities. | * Professional identity formation: Not available in metadata. * Ethical reasoning: Present (“ethical implications”). * Moral agency: Not available in metadata. * Clinical judgment in algorithmic contexts: Present (“clinical judgment” relevance is “Somewhat Relevant”). * Digital professionalism: Present (“digital competence,” “Digital Professionalism” relevance is “Highly Relevant”). * Power/bias awareness: Present (“bias” in abstract, “Algorithmic Bias” relevance is “Highly Relevant”). * Recognition that AI mediates clinical decisions: Not available in metadata. * Preparation for algorithmic practice: Not available in metadata. | Generative AI, such as ChatGPT, offers promising potential for nursing education through personalized learning, simulation training, and teaching process support. However, its integration necessitates careful consideration of ethical implications, curriculum adaptation, and high digital competence among teachers. This approach is crucial to mitigate risks like knowledge distortion, bias, and educational inequalities. |
| 3. | Laura González | 2024 | Nursing Education in the Era of ChatGPT: Implications and Opportunities | Commentary/editorial | - Primary AI Focus: AI in simulation/education - Specific AI Technologies Mentioned: ChatGPT, generative pre-trained transformer language model | * professional identity formation: Not available in metadata. * ethical reasoning: Discussed through “ethical concerns related to AI,” “biases, data privacy, transparency, and other psychosocial concerns,” and “cautious and ethical use of AI.” * moral agency: Not available in metadata. * clinical judgment in algorithmic contexts: Not directly mentioned, but implied by “equipping future nurses with digital and data literacy skills necessary for working in today’s increasingly AI-driven environments.” * digital professionalism: Not explicitly mentioned, but “digital and data literacy skills” are highlighted. * power/bias awareness: Discussed as “biases” within ethical concerns. * recognition that AI mediates clinical decisions: Implied by “working in today’s increasingly AI-driven environments” and the focus on “implications and opportunities” of ChatGPT in nursing education. * preparation for algorithmic practice: Implied by “equipping future nurses with digital and data literacy skills necessary for working in today’s increasingly AI-driven environments.” | This article explores the implications of ChatGPT in nursing education, emphasizing benefits like enhanced teaching, critical thinking, and personalized learning. It also addresses barriers, ethical concerns, and provides recommendations for responsible AI integration. The work underscores the importance of transforming nursing curricula to equip future nurses with necessary digital and data literacy skills. |
| 4. | Rachel Cox Simms | 2024 | Work With ChatGPT, Not Against: 3 Teaching Strategies That Harness the Power of Artificial Intelligence. | Commentary/editorial | * Primary AI focus: AI in simulation/education * Specific AI technologies mentioned: ChatGPT, generative AI | * professional identity formation: Not available in metadata. * ethical reasoning: Not available in metadata. * moral agency: Not available in metadata. * clinical judgment in algorithmic contexts: Present, as the approach reinforces clinical judgment and prepares students for AI-prevalent health care environments. * digital professionalism: Not available in metadata. * power/bias awareness: Not available in metadata. * recognition that AI mediates clinical decisions: Present, as the article addresses challenges to academic integrity, validating information accuracy, and ensuring credibility of AI-generated information, and prepares students for AI-prevalent health care environments. * preparation for algorithmic practice: Present, as the approach involves practical activities for students to engage with AI-generated content critically, preparing them for AI-prevalent health care environments. | The article concludes that incorporating AI tools such as ChatGPT into nursing curricula is a strategic educational advancement, equipping students with essential skills to navigate modern health care. This approach involves practical activities for students to critically engage with AI-generated content, reinforcing clinical judgment and preparing them for AI-prevalent health care environments. It highlights the need for strategies to ensure information accuracy and credibility while enhancing learning through critical evaluation. |
| 5. | Andy Hermógenes Luque Loor et al. | 2025 | Implications of Generative Artificial Intelligence on Ethics in Nursing Education: State of The Art Review. | state-of-the-art review | The primary AI focus is AI in simulation/education and AI ethics/bias. The paper specifically mentions Generative Artificial Intelligence (GAI). | * Ethical reasoning: Present. The study focuses on ethical implications, degradation of ethical reasoning is a concern, and ethical training is essential. * Clinical judgment in algorithmic contexts: Present. GAI offers opportunities for improving clinical reasoning, and safeguarding professional judgment is mentioned. * Power/bias awareness: Present. Bias mitigation is highlighted as a need. * Recognition that AI mediates clinical decisions: Present. GAI has the potential to enhance clinical decision-making. * Preparation for algorithmic practice: Present. The paper discusses pedagogical strategies and hybrid pedagogical approaches. * Professional identity formation: Not available in metadata. * Moral agency: Not available in metadata. * Digital professionalism: Not available in metadata. | The integration of Generative AI in nursing education offers opportunities to improve clinical reasoning and knowledge among students, despite raising concerns about academic integrity, automation bias, and data privacy. To manage ethical risks like the degradation of ethical reasoning and issues of privacy and equity, clear governance frameworks, hybrid pedagogical approaches with “human-in-the-loop,” and explicit guidelines for transparency, bias mitigation, and data protection are essential. A hybrid design approach, coupled with ethical training and continuous evaluation of AI use, is crucial to ensure GAI’s benefits while safeguarding professional judgment and patient safety. |
| 6. | Shu‐Ling Chen & Lin‐Lin Lee | 2024 | [Strengths, Weaknesses, Opportunities, and Threats Analysis of Integrating ChatGPT Into Nursing Education]. | Commentary/editorial | AI in simulation/education: AI language models such as ChatGPT hold the potential to revolutionize the delivery of nursing education. AI ethics/bias: The technology has limitations and potential biases. AI literacy: Faculty need to be familiar with capabilities and limitations to foster effective, ethical, and responsible utilization of AI technology. Specific AI technologies mentioned: ChatGPT, generative AI. | * professional identity formation: Not explicitly present. * ethical reasoning: Present (foster effective, ethical, and responsible utilization of AI technology). * moral agency: Not explicitly present. * clinical judgment in algorithmic contexts: Not explicitly present. * digital professionalism: Not explicitly present. * power/bias awareness: Present (technology has limitations and potential biases). * recognition that AI mediates clinical decisions: Not explicitly present. * preparation for algorithmic practice: Present (preparing students in advance for the dynamic and rapidly advancing landscape of nursing and healthcare; prepare nursing students for a future technology-integrated healthcare system). | This study conducts a SWOT analysis of integrating ChatGPT into nursing education, highlighting its potential to revolutionize delivery while also addressing its limitations, biases, and challenges. It provides a guide for nurse educators to make informed decisions about AI technology implementation. The article aims to help faculty understand ChatGPT’s capabilities and limitations to foster effective, ethical, and responsible utilization of AI in preparing students for a technology-integrated healthcare system. |
| 7. | Jennie C. De Gagne et al. | 2023 | Cyberethics in nursing education: Ethical implications of artificial intelligence. | Commentary/editorial | - AI in simulation/education - AI ethics/bias - Specific AI technologies mentioned: artificial intelligence (AI), generative AI (Gen AI) | * ethical reasoning: Present. The article highlights ethical principles (autonomy, nonmaleficence, beneficence, justice, explicability) as a roadmap for AI integration and notes that ethical dilemmas can emerge. It emphasizes that adherence to these principles offers solutions. * digital professionalism: Present. The article discusses “cyberethics,” which is a field of applied ethics focusing on the ethical, legal, and social implications of cybertechnology. * power/bias awareness: Not explicitly present in metadata. * recognition that AI mediates clinical decisions: Not explicitly present in metadata, though the context is AI in nursing education for future patient care. * preparation for algorithmic practice: Present. The article states that nurse educators have a pivotal role in strategizing approaches for ethical AI integration and ensuring future nurses can navigate evolving healthcare technology. * professional identity formation: Not explicitly present in metadata. * moral agency: Not explicitly present in metadata. * clinical judgment in algorithmic contexts: Not explicitly present in metadata. | The article highlights that cyberethics principles—autonomy, nonmaleficence, beneficence, justice, and explicability—serve as a roadmap for integrating AI into nursing education, addressing potential ethical dilemmas. It recommends weaving these principles into curricula and developing appropriate guidelines to ensure ethical and responsible use of generative AI. Nurse educators are crucial in strategizing ethical AI integration, establishing clear guidelines, and fostering critical thinking and adaptability among students. |
| 8. | Siah et al. | 2026 | Authenticity and academic integrity in Generative Artificial Intelligence (GenAI) use among undergraduate nursing students. | Other. | - Primary AI focus: AI in simulation/education, AI ethics/bias. - Specific AI technologies mentioned: Generative Artificial Intelligence (GenAI), large Generative Artificial Intelligence (GenAI). | * professional identity formation: The metadata mentions “Student Identity Development” as “Somewhat Relevant”. * ethical reasoning: Listed as “Ethical Reasoning” and rated “Highly Relevant”. * moral agency: Not available in metadata. * clinical judgment in algorithmic contexts: “Clinical Judgment” is listed as “Not Relevant”. * digital professionalism: Listed as “Digital Professionalism” and rated “Highly Relevant”. * power/bias awareness: “Algorithmic Bias” is listed as “Not Relevant”. * recognition that AI mediates clinical decisions: Not available in metadata. * preparation for algorithmic practice: Not available in metadata. | The use of Generative Artificial Intelligence (GenAI) in higher education is reshaping student learning, offering benefits but also posing ethical challenges. It may impact students’ ability to identify learning gaps and engage with course content. The paper highlights the importance of authenticity and academic integrity in GenAI use among undergraduate nursing students. |
| 9. | T. C. Fung et al. | 2025 | Effects of generative artificial intelligence (GenAI) patient simulation on clinical competency among global nursing undergraduates: A cross-over randomised controlled trial | empirical study (cross-over randomised controlled trial) | AI in simulation/education, specifically Generative Artificial Intelligence (GenAI) patient simulation. | * professional identity formation: Not available in metadata. * ethical reasoning: Not available in metadata. * moral agency: Not available in metadata. * clinical judgment in algorithmic contexts: Not available in metadata. * digital professionalism: Not available in metadata. * power/bias awareness: Not available in metadata. * recognition that AI mediates clinical decisions: Not available in metadata. * preparation for algorithmic practice: Not available in metadata. | The study found that both 360° VR simulation and GenAI patient simulation effectively improved nursing students’ clinical outcomes, including clinical competence, cultural awareness, and AI readiness. GenAI patient simulation, particularly with real-time feedback and GenAI debriefing, demonstrated a notably greater effect on these measures. These findings suggest that GenAI can serve as a powerful teaching tool for enhancing clinical competency in nursing education. |
| 10. | J Vanderlaan et al. | N/A | Practical Intelligence: Generative AI Toolkit for Nurse Education | guideline/toolkit | * Primary AI Focus: AI in simulation/education, multiple AI applications. * Specific AI Technologies Mentioned: Generative AI models. | * Professional Identity Formation: Highly Relevant (Professional Formation) * Ethical Reasoning: Somewhat Relevant * Moral Agency: Not available in metadata. * Clinical Judgment in Algorithmic Contexts: Highly Relevant (Clinical Judgment) * Digital Professionalism: Somewhat Relevant * Power/Bias Awareness: Somewhat Relevant (Algorithmic Bias) * Recognition that AI mediates clinical decisions: Not available in metadata. * Preparation for Algorithmic Practice: Not available in metadata. | The paper utilizes a generative AI toolkit to design nursing curricula, support clinical simulations, and provide AI-generated feedback. It also discusses professional formation, addresses integrity, ethics, bias, and implementation hurdles in nurse education. |
| 11. | Hwang & Yu | 2026 | From film to simulation: Mixed-methods study of learner-driven artificial intelligence-generated ethical dilemma scenarios and role-play to enhance ethical competence in nursing students. | empirical study | * Primary AI Focus: AI in simulation/education * Specific AI Technologies: Artificial Intelligence (AI)-Generated ethical dilemma scenarios | * Professional Identity Formation: Not available in metadata. (The relevance metadata indicates “Student Identity Development” is “Not Relevant”.) * Ethical Reasoning: Present. The study aims to enhance ethical competence and biomedical ethics awareness. * Moral Agency: Not available in metadata. * Clinical Judgment in Algorithmic Contexts: Not available in metadata. (The relevance metadata indicates “Clinical Judgment” is “Somewhat Relevant” but doesn’t specify algorithmic contexts.) * Digital Professionalism: Not available in metadata. (The relevance metadata indicates “Digital Professionalism” is “Not Relevant”.) * Power/Bias Awareness: Not available in metadata. (The relevance metadata indicates “Algorithmic Bias” is “Not Relevant”.) * Recognition that AI mediates clinical decisions: Not available in metadata. * Preparation for algorithmic practice: Not available in metadata. | The study aimed to develop and implement a Film-Based, AI-Generated Interactive Ethical Dilemma Role-Play Simulation program and evaluate its effects on nursing students. This program is intended to enhance nursing students’ biomedical ethics awareness, critical thinking disposition, and empathy. The research also seeks to gather data on program satisfaction, suggesting implications for novel teaching methods in nursing education. |
| 12. | Hardie et al. | 2026 | Applications, attitudes and ethical considerations of Generative Artificial Intelligence (Gen AI) in nursing education: a scoping review. | scoping review | The primary AI focus is multiple AI applications, specifically Generative Artificial Intelligence (Gen AI) in nursing education. Specific AI technologies mentioned include: * Generative Artificial Intelligence (Gen AI) * ChatGPT | * Professional identity formation: Not explicitly mentioned. * Ethical reasoning: Highly Relevant. * Moral agency: Not explicitly mentioned. * Clinical judgment in algorithmic contexts: Somewhat Relevant (as “Clinical Judgment”). * Digital professionalism: Somewhat Relevant. * Power/bias awareness: Somewhat Relevant (as “Algorithmic Bias”). * Recognition that AI mediates clinical decisions: Not explicitly mentioned. * Preparation for algorithmic practice: Not explicitly mentioned. | This scoping review examines the applications, attitudes, and ethical considerations of Generative Artificial Intelligence (Gen AI) in nursing education. It highlights how Gen AI technologies are increasingly integrated to support various pedagogical activities within the field. The review aims to map Gen AI uses, student views, academic integrity, bias, pedagogical strategies, and implementation challenges, along with ethical reasoning and clinical practice preparation. |
| 13. | Anthony Summers et al. | 2024 | Navigating challenges and opportunities: Nursing student’s views on generative AI in higher education | empirical study | AI in simulation/education: The study explores nursing students’ perspectives on the application and integration of generative Artificial Intelligence (AI) tools in their studies and education. Specific AI technologies mentioned: Generative AI. | * professional identity formation: Not available in metadata. * ethical reasoning: Not available in metadata. * moral agency: Not available in metadata. * clinical judgment in algorithmic contexts: Not available in metadata. * digital professionalism: Not available in metadata. * power/bias awareness: Not available in metadata. * recognition that AI mediates clinical decisions: Not available in metadata. * preparation for algorithmic practice: The study explores nursing students’ views on the application and integration of generative AI tools in their studies, and the potential for both positive contributions and challenges associated with the integration of generative AI in nursing education and practice. | This initial exploration reveals diverse perspectives of nursing students regarding generative AI tools in their education. It highlights both potential positive contributions and challenges associated with integrating generative AI in nursing education and practice. |
| 14. | VC Chan & AL Hequembourg | N/A | Application of generative artificial intelligence in undergraduate nursing education: A scoping review | scoping review | Primary AI focus: AI in simulation/education. Specific AI technologies mentioned: - Generative AI (genAI) | * Professional identity formation: Not available in metadata. * Ethical reasoning: Somewhat Relevant (as “Ethical Reasoning”). * Moral agency: Not available in metadata. * Clinical judgment in algorithmic contexts: Somewhat Relevant (as “Clinical Judgment”). * Digital professionalism: Somewhat Relevant (as “Digital Professionalism”). * Power/bias awareness: Highly Relevant (as “Algorithmic Bias”). * Recognition that AI mediates clinical decisions: Not available in metadata. * Preparation for algorithmic practice: Highly Relevant (as “Clinical Practice Preparation”). | A scoping review mapped the uses of generative AI in undergraduate nursing programs, including its integration, potential for bias, and impact on academic integrity and simulation tools. The integration of generative AI in nursing education demands attention to concerns around bias and academic integrity. |
| 15. | Yang et al. | 2026 | A large language model-powered reflective AI agent for evidence-based nursing education: Design and evaluation. | empirical study | AI in simulation/education: large language model-powered reflective AI agent. | - Professional identity formation: Highly Relevant (Professional Formation) and Somewhat Relevant (Student Identity Development). - Ethical reasoning: Somewhat Relevant. - Moral agency: Not available in metadata. - Clinical judgment in algorithmic contexts: Highly Relevant (Clinical Judgment) and the paper uses a large language model-powered AI agent. - Digital professionalism: Not Relevant. - Power/bias awareness: Not Relevant (Algorithmic Bias). - Recognition that AI mediates clinical decisions: The paper focuses on an AI agent for education, not direct mediation of clinical decisions. - Preparation for algorithmic practice: Enhances clinical judgment and practice preparation, using an LLM-driven reflective agent. | The paper focuses on developing and evaluating EBN-Expert, a large language model-powered reflective AI agent designed to support reflective learning in evidence-based nursing education. This agent enhances clinical judgment and prepares students for practice by integrating generative AI into nursing education. The study also discusses curriculum design and evaluation for this purpose. |
| 16. | Zhengxin Ni et al. | 2024 | Embracing the future: Integrating ChatGPT into China’s nursing education system | Commentary/editorial | AI in simulation/education, ChatGPT, Large Language Models (LLMs) | * Professional identity formation: Not available in metadata. * Ethical reasoning: Not available in metadata. * Moral agency: Not available in metadata. * Clinical judgment in algorithmic contexts: The paper discusses ChatGPT’s potential in nursing education and addressing clinical issues, and its performance on the National Nursing Licensure Examination, implying interaction with algorithmic contexts. * Digital professionalism: Not available in metadata. * Power/bias awareness: Not available in metadata. * Recognition that AI mediates clinical decisions: The paper highlights ChatGPT’s role in facilitating learning and addressing clinical issues, and its application in clinical settings, indicating AI’s mediation in decisions. * Preparation for algorithmic practice: The paper discusses integrating ChatGPT into nursing education and its application in clinical settings, suggesting preparation for algorithmic practice. | ChatGPT shows significant potential in China’s nursing education due to its interactive learning and real-time feedback capabilities, and its higher accuracy rate on China’s National Nursing Licensure Examination compared to other LLMs. To realize its full potential, educational institutions should establish a targeted regulatory framework, provide standardized training, and adjust assessment methods to prevent misuse. This integration aligns with modern teaching methodologies and addresses the developmental needs of nursing education. |
| 17. | Yoon et al. | 2026 | The potential impact of generative AI across Miller’s pyramid of clinical competence: A systematic review. | Systematic review | Primary AI focus: AI in simulation/education. Specific AI technologies mentioned: Generative artificial intelligence (GenAI). | - Professional identity formation: Present (listed as ‘Student Identity Development’ with ‘Somewhat Relevant’). - Ethical reasoning: Present (listed as ‘Ethical Reasoning’ with ‘Somewhat Relevant’). - Moral agency: Not available in metadata. - Clinical judgment in algorithmic contexts: Present (listed as ‘Clinical Judgment’ with ‘Highly Relevant’). - Digital professionalism: Absent (listed as ‘Digital Professionalism’ with ‘Not Relevant’). - Power/bias awareness: Absent (listed as ‘Algorithmic Bias’ with ‘Not Relevant’). - Recognition that AI mediates clinical decisions: Not available in metadata. - Preparation for algorithmic practice: Not available in metadata. | This systematic review explores the effectiveness and limitations of generative AI in developing clinical competence within nursing education, aiming to inform the design of educator-guided, Expert-in-the-Loop hybrid educational frameworks. It synthesizes evidence to map competency development and clinical preparation, touching on identity, ethics, and perceptions. The review’s findings are intended to guide teaching frameworks in this evolving field. |
| 18. | Daniel Rodrigues & R. Cruz-Correia | 2024 | Large Language Models in Nursing Education: State-of-the-Art. | Literature review | AI in simulation/education: Large Language Models (LLMs), ChatGPT. | * Professional identity formation: Not available in metadata. * Ethical reasoning: Present (ethical use). * Moral agency: Not available in metadata. * Clinical judgment in algorithmic contexts: Present (simulate complex clinical scenarios). * Digital professionalism: Not available in metadata. * Power/bias awareness: Not available in metadata. * Recognition that AI mediates clinical decisions: Not available in metadata. * Preparation for algorithmic practice: Not available in metadata. | This study highlights that Large Language Models (LLMs) offer unique opportunities to enhance curriculum delivery, foster critical thinking, and simulate complex clinical scenarios in nursing education, enabling interactive learning environments. The paper navigates the complexities of adopting LLMs and concludes with a call to action to advance AI integration in nursing. This integration aims to enhance educational outcomes while ensuring ethical and effective use. |
| 19. | LJ Labrague | N/A | A five-tier framework for guiding responsible AI use in nursing students’ coursework: A faculty guide | Commentary/editorial | * Primary AI focus: ChatGPT/generative AI as writing tool, AI in simulation/education, AI ethics/bias, AI literacy. * Specific AI technologies mentioned: ChatGPT. | * professional identity formation: Not available in metadata. * ethical reasoning: Highly Relevant. * moral agency: Not available in metadata. * clinical judgment in algorithmic contexts: Not available in metadata. * digital professionalism: Highly Relevant. * power/bias awareness: Somewhat Relevant (Algorithmic Bias). * recognition that AI mediates clinical decisions: Not available in metadata. * preparation for algorithmic practice: Not available in metadata. | The paper presents a five-tier framework designed to guide the responsible use of AI in nursing students’ coursework. This framework aims to address critical aspects such as academic integrity, ethical reasoning, and digital professionalism within nursing education. It also offers pedagogical guidance for faculty and touches upon issues like algorithmic bias and implementation challenges. |
| 20. | D Stratton-Maher | N/A | The Impact of Generative AI on Portfolio Assessment in the Bachelor of Nursing Program at the University of Southern Queensland | This PDF is an eBook of Shortened Peer Reviewed Papers, meaning it contains multiple distinct papers. Here are the classifications for each paper: * Paper 1: Integrating ePortfolios in a Master of Science core course hybrid environment * Empirical study (p. 3 states “present preliminary results detailing the impact of the changes.”) * Paper 2: Create@UniSQ: Initiating a university wide, flexible and authentic student portfolio program for real world learning. * Theoretical paper / Case study (describes the development and implementation of a program, with qualitative evidence and survey feedback, but not a formal empirical study with quantitative results, p. 9-10). * Paper 3: The Impact of Generative AI on Portfolio Assessment in the Bachelor of Nursing Program at the University of Southern Queensland * Theoretical paper / Commentary (explores the role and potential of GenAI in nursing education, discusses synergy and reimagining portfolios, but does not present new empirical data, p. 17-20). * Paper 4: Using generative AI to enhance ePortfolio practices * Theoretical paper / Commentary (explores conceptual applications of an AI model to ePortfolio practices, offering guidance and suggestions, p. 23-27). * Paper 5: Programmatic assessment design using eportfolio from concept, design and use in Dentistry * Case study / Empirical study (details a specific programmatic assessment design, presents grade distribution data and student feedback, p. 30-36). * Paper 6: Australian and New Zealand ePortfolio Research: Implications for Future Practice in Tertiary Education * Empirical study (presents findings from a survey of 39 staff members across Australian and New Zealand tertiary institutions, p. 38-46). * Paper 7: Developing Future Educators: The Role of ePortfolios in a Graduate Teaching Associates Program * Case study (describes the integration of an ePortfolio into a specific program, p. 51-55). * Paper 8: Developing independent responsibility for learning within the Pharmacy Intern Training Program * Case study (describes how an ePortfolio is used in a specific program, includes figures showing intern progress, p. 57-66). | The primary AI focus is multiple AI applications, with a strong emphasis on ChatGPT/generative AI as a writing tool and AI in simulation/education. Specific AI technologies mentioned include: * Generative AI (GenAI): Used for enhancing ePortfolio practices, portfolio assessment in nursing, generating content, providing personalized feedback, creating immersive learning environments, and academic writing support. * ChatGPT: Specifically used as a writing tool for structuring language and crafting reflective writing pieces in nursing education. | Here are the professional formation elements identified in the paper: * Professional Identity Formation: * Present. The Create@UniSQ program aims to foster professional identity (page 9). In the Bachelor of Nursing program, portfolios play a crucial role in shaping professional identity (page 18). Generative AI (GenAI) can impact professional identity formation by providing customized feedback and support (page 20). The Pharmacy Intern Program (ITP) helps interns develop a learning plan to address knowledge gaps, aligning practice with continuing professional development (page 57, 63). * Ethical Reasoning: * Present. The paper mentions ensuring the ethical use of AI and adherence to academic integrity guidelines in the context of GenAI in nursing education (page 19). Practical guidance on ethical implementation of AI technologies was emphasized (page 19). The AAEEBL Digital Ethics in ePortfolios Task Force established ethical principles for ePortfolios (page 40). * Moral Agency: * Not addressed in this paper. * Clinical Judgment in Algorithmic Contexts: * Limited. The paper discusses GenAI’s ability to simulate complex clinical scenarios in nursing education (page 19) and prepare students for healthcare complexities (page 18), implying an interaction with clinical judgment, but does not explicitly detail “clinical judgment in algorithmic contexts.” * Digital Professionalism: * Not explicitly addressed in this paper, though “digital literacy” and “professional identity” are mentioned in relation to ePortfolios (page 11, 18). * Power/Bias Awareness: * Not addressed in this paper. * Recognition that AI mediates clinical decisions: * Limited. The paper states that GenAI can simulate complex clinical scenarios and prepare the nursing workforce with relevant skills (page 19), and that AI-driven tools can enhance educational experience (page 19). However, it does not explicitly state recognition that AI mediates clinical decisions. * Preparation for algorithmic practice: * Present. The use of GenAI in nursing education is discussed as preparing students for healthcare complexities and emphasizing technological fluency in contemporary practice (page 18, 19). | The integration of Generative AI (GenAI) with portfolio assessments in the Bachelor of Nursing program at the University of Southern Queensland has created a transformative learning environment, merging traditional educational values with progressive technology. This synergy enhances educational effectiveness and prepares nursing students for healthcare complexities, with GenAI facilitating personalized learning pathways, tailored writing assistance, and immediate feedback. The dynamic nature of GenAI-facilitated portfolios sets a new standard in nursing education, equipping students with essential skills and experiences that enhance their employability. |
| 21. | Go & Lee | 2026 | The effectiveness of AI-based conversational agents in nursing education: A systematic review. | Systematic review | AI in simulation/education; Specific AI technologies: AI-based conversational agents (chatbots, virtual assistants) | * Professional identity formation: Not addressed. * Ethical reasoning: Not addressed. * Moral agency: Not addressed. * Clinical judgment: Present — conversational agents support clinical reasoning development. * Digital professionalism: Not addressed. * Power awareness: Not addressed. * Recognition of clinical AI infrastructures: Not addressed. | Systematic review of AI-based conversational agents in nursing education. Finds evidence that AI chatbots and virtual assistants can support knowledge acquisition and clinical reasoning, but highlights variability in implementation quality and limited evidence on long-term outcomes. |
| 22. | N Hilsmann & C Dodson | N/A | WNRCASN Conference 2025 | Conference paper | Multiple AI applications in nursing education | * Professional identity formation: Not available in metadata. * Ethical reasoning: Not available in metadata. * Moral agency: Not available in metadata. * Clinical judgment: Not available in metadata. * Digital professionalism: Not available in metadata. * Power awareness: Not available in metadata. * Recognition of clinical AI infrastructures: Not available in metadata. | Conference presentation (WNRCASN 2025). Limited metadata available. Focus on AI integration in nursing education contexts within Western and Northern Regional Council of Associate Science in Nursing programs. |
| 23. | Maxim Topaz et al. | 2024 | The ChatGPT Effect: Nursing Education and Generative Artificial Intelligence. | Commentary/editorial | ChatGPT/generative AI as writing tool and educational technology; specific AI: ChatGPT | * Professional identity formation: Present — discusses how ChatGPT reshapes nursing professional identity. * Ethical reasoning: Present — ethical implications of AI use in education discussed. * Moral agency: Not addressed. * Clinical judgment: Not addressed. * Digital professionalism: Present — digital literacy and responsible AI use foregrounded. * Power awareness: Not addressed. * Recognition of clinical AI infrastructures: Not addressed. | Examines the ‘ChatGPT Effect’ on nursing education and generative AI adoption. Argues nursing education must adapt curricula to address generative AI’s disruptive impact, emphasising both opportunities for learning enhancement and risks to academic integrity and professional identity formation. |
| 24. | Youzhu Yuan et al. | 2025 | The strengths, weaknesses, opportunities, and threats of generative artificial intelligence: a qualitative study of undergraduate nursing students | Empirical study (qualitative) | ChatGPT/generative AI; SWOT analysis of generative AI from nursing student perspectives | * Professional identity formation: Not addressed. * Ethical reasoning: Present — ethical concerns identified as a threat in SWOT analysis. * Moral agency: Not addressed. * Clinical judgment: Present — clinical reasoning implications discussed. * Digital professionalism: Present — digital competency as opportunity. * Power awareness: Not addressed. * Recognition of clinical AI infrastructures: Not addressed. | Qualitative SWOT analysis with undergraduate nursing students on generative AI. Strengths include enhanced learning efficiency; weaknesses include over-reliance and reduced critical thinking; opportunities include personalised learning; threats include academic dishonesty and erosion of ethical reasoning. |
| 25. | M Gerdes et al. | N/A | Emerging artificial intelligence-based pedagogies in didactic nursing education: a scoping review | Scoping review | Multiple AI applications; AI-based pedagogies in nursing didactic education | * Professional identity formation: Not addressed. * Ethical reasoning: Not addressed. * Moral agency: Not addressed. * Clinical judgment: Present — AI pedagogies aimed at clinical reasoning. * Digital professionalism: Present — digital competency development. * Power awareness: Not addressed. * Recognition of clinical AI infrastructures: Not addressed. | Scoping review of emerging AI-based pedagogical approaches in didactic nursing education. Maps the landscape of AI tools used in classroom and online settings, identifying key gaps in evidence and calls for more rigorous evaluation of educational outcomes. |
| 26. | Isaac Amankwaa et al. | 2025 | Patterns, Advances, and Gaps in Using ChatGPT and Similar Technologies in Nursing Education: A PAGER Scoping Review | Scoping review (PAGER framework) | ChatGPT/generative AI; patterns, advances, and gaps in ChatGPT use in nursing education | * Professional identity formation: Not addressed. * Ethical reasoning: Present — academic integrity and ethical AI use discussed. * Moral agency: Not addressed. * Clinical judgment: Not addressed. * Digital professionalism: Present — AI literacy and responsible use. * Power awareness: Not addressed. * Recognition of clinical AI infrastructures: Not addressed. | PAGER scoping review of ChatGPT and similar technologies in nursing education. Identifies patterns of use (assessment assistance, study support, clinical case generation), key advances in AI-assisted learning, and significant gaps including lack of faculty training, policy frameworks, and equity considerations. |
| 27. | Myung Jin Choi et al. | 2025 | Research trends in generative artificial intelligence in nursing: a scoping review | Scoping review | Multiple AI applications; generative AI research trends in nursing broadly | * Professional identity formation: Not addressed. * Ethical reasoning: Present — ethical considerations in AI use noted. * Moral agency: Not addressed. * Clinical judgment: Present — clinical AI applications included. * Digital professionalism: Not addressed. * Power awareness: Not addressed. * Recognition of clinical AI infrastructures: Present — clinical AI infrastructure referenced. | Scoping review mapping research trends in generative AI in nursing across education and clinical practice. Finds rapid growth in publications post-2022, with education and clinical decision support as dominant themes. Highlights need for evidence-based frameworks and ethical guidelines. |
| 28. | Ramteja Sajja et al. | 2025 | Evaluating artificial intelligence assisted nursing education: Student perceptions, ethical concerns, and pedagogical implications | Empirical study (mixed methods/survey) | AI in simulation/education; student perceptions of AI-assisted nursing education | * Professional identity formation: Not addressed. * Ethical reasoning: Present — ethical concerns about AI in education surveyed. * Moral agency: Not addressed. * Clinical judgment: Not addressed. * Digital professionalism: Present — AI literacy implications discussed. * Power awareness: Not addressed. * Recognition of clinical AI infrastructures: Not addressed. | Evaluates AI-assisted nursing education from student perspectives. Finds generally positive perceptions of AI tools for learning support, but significant ethical concerns about academic integrity, bias, and over-reliance. Calls for pedagogical frameworks that balance AI benefits with critical engagement. |
| 29. | Jason Blomquist et al. | 2025 | Student and Faculty Perceptions and Use of Generative Artificial Intelligence in Nursing Education | Empirical study (survey) | ChatGPT/generative AI; student and faculty perceptions and use in nursing education | * Professional identity formation: Not addressed. * Ethical reasoning: Present — ethical concerns about AI use discussed. * Moral agency: Not addressed. * Clinical judgment: Not addressed. * Digital professionalism: Present — responsible AI use and digital literacy. * Power awareness: Not addressed. * Recognition of clinical AI infrastructures: Not addressed. | Survey of nursing students and faculty on perceptions and actual use of generative AI tools. Finds widespread use among students with limited formal guidance; faculty express concerns about academic integrity and lack of institutional policy. Highlights generational and role-based differences in AI adoption. |
| 30. | Stratton-Maher & Kelly | 2026 | Learning with AI, not from AI: Ethical and pedagogical implications of ChatGPT in undergraduate education. | Commentary/editorial | ChatGPT/generative AI; ethical and pedagogical implications of ChatGPT in undergraduate education | * Professional identity formation: Not addressed. * Ethical reasoning: Present — central focus on ethical use of AI in learning. * Moral agency: Present — student moral responsibility in AI use discussed. * Clinical judgment: Not addressed. * Digital professionalism: Present — responsible digital citizenship. * Power awareness: Not addressed. * Recognition of clinical AI infrastructures: Not addressed. | Argues for a pedagogy of ‘learning with AI, not from AI’ — positioning generative AI as a collaborative tool rather than a knowledge source. Emphasises ethical engagement, critical thinking, and the development of moral agency in students navigating AI-assisted learning environments. |
| 31. | Molu | 2025 | Nursing students’ perceptions of gender and race in AI healthcare imagery. | Empirical study (survey/content analysis) | AI ethics/bias; nursing students’ perceptions of gender and race representation in AI healthcare imagery | * Professional identity formation: Present — explores how AI imagery shapes professional identity perceptions. * Ethical reasoning: Present — bias and equity in AI representations. * Moral agency: Not addressed. * Clinical judgment: Not addressed. * Digital professionalism: Not addressed. * Power awareness: Present — power dynamics in AI-generated representations of healthcare workers. * Recognition of clinical AI infrastructures: Not addressed. | Examines nursing students’ critical perceptions of gender and racial representation in AI-generated healthcare imagery. Finds students notice and are troubled by stereotypical representations, raising concerns about how AI reinforces existing professional hierarchies and identity norms in nursing. |
| 32. | Phillip Olla et al. | 2024 | Promptology: Enhancing Human-AI Interaction in Large Language Models | Commentary/editorial | Multiple AI applications; LLMs; human-AI interaction and prompt engineering | * Professional identity formation: Not addressed. * Ethical reasoning: Not addressed. * Moral agency: Not addressed. * Clinical judgment: Not addressed. * Digital professionalism: Present — prompt engineering as a professional AI competency. * Power awareness: Not addressed. * Recognition of clinical AI infrastructures: Not addressed. | Introduces ‘promptology’ as a framework for enhancing human-AI interaction with large language models. Proposes structured approaches to prompt engineering that improve AI output quality and relevance, with applications across healthcare and educational contexts. |
| 33. | P Hodge | N/A | Healthcare Educators NET Conference 2025-Sustainability of Healthcare Education: Enabling the Future | Conference paper | Multiple AI applications in healthcare education; sustainability of healthcare education | * Professional identity formation: Not available in metadata. * Ethical reasoning: Not available in metadata. * Moral agency: Not available in metadata. * Clinical judgment: Not available in metadata. * Digital professionalism: Not available in metadata. * Power awareness: Not available in metadata. * Recognition of clinical AI infrastructures: Not available in metadata. | Conference presentation (Healthcare Educators NET 2025). Focus on sustainability of healthcare education in the context of emerging AI technologies. Limited metadata available for full extraction. |
| 34. | M Al-Hassan & R Dorri | N/A | Harnessing AI for nursing education: innovations, barriers, and ethical considerations | Literature review | Multiple AI applications; innovations, barriers, and ethical considerations in AI for nursing education | * Professional identity formation: Not addressed. * Ethical reasoning: Present — ethical considerations as a core theme. * Moral agency: Not addressed. * Clinical judgment: Not addressed. * Digital professionalism: Present — digital competency and AI literacy. * Power awareness: Not addressed. * Recognition of clinical AI infrastructures: Not addressed. | Reviews innovations in AI for nursing education alongside barriers to adoption and ethical considerations. Identifies institutional resistance, lack of faculty training, and ethical frameworks as key challenges. Recommends structured AI integration policies and faculty development programs. |
| 35. | E Larsen & K Burke | N/A | V102. | Conference paper | AI in nursing education (specific focus not available from metadata) | * Professional identity formation: Not available in metadata. * Ethical reasoning: Not available in metadata. * Moral agency: Not available in metadata. * Clinical judgment: Not available in metadata. * Digital professionalism: Not available in metadata. * Power awareness: Not available in metadata. * Recognition of clinical AI infrastructures: Not available in metadata. | Conference paper (V102). Limited metadata available. Specific focus and findings not determinable from available bibliographic information. |
| 36. | Emily Tomlinson et al. | 2024 | A Course-Wide Approach to Building Generative Artificial Intelligence Literacy Across an Undergraduate Nursing Curriculum | Empirical study (curriculum development/descriptive) | ChatGPT/generative AI; AI literacy integration across undergraduate nursing curriculum | * Professional identity formation: Not addressed. * Ethical reasoning: Present — ethical AI use embedded in curriculum approach. * Moral agency: Not addressed. * Clinical judgment: Not addressed. * Digital professionalism: Present — AI literacy as core curricular competency. * Power awareness: Not addressed. * Recognition of clinical AI infrastructures: Not addressed. | Describes a course-wide approach to building generative AI literacy across an undergraduate nursing curriculum. Reports on structured scaffolding of AI competencies from introductory to advanced levels, with emphasis on critical evaluation of AI outputs and ethical use in academic and clinical contexts. |
| 37. | Susan Hayes Lane et al. | 2024 | Tool or Tyrant: Guiding and Guarding Generative Artificial Intelligence Use in Nursing Education. | Commentary/editorial | ChatGPT/generative AI; guiding and guarding AI use in nursing education | * Professional identity formation: Not addressed. * Ethical reasoning: Present — ethical boundaries of AI use in education. * Moral agency: Present — student responsibility in AI use. * Clinical judgment: Not addressed. * Digital professionalism: Present — responsible AI use as professional skill. * Power awareness: Not addressed. * Recognition of clinical AI infrastructures: Not addressed. | Positions generative AI as both a ‘tool’ and a potential ‘tyrant’ in nursing education. Argues for structured guidance frameworks that harness AI benefits while guarding against academic dishonesty, over-reliance, and erosion of critical thinking. Proposes educator-led AI governance approaches. |
| 38. | Hall et al. | 2026 | End-of-life education: An explorative study using artificial intelligence simulations in undergraduate nursing. | Empirical study (exploratory/qualitative) | AI in simulation/education; AI simulations for end-of-life education in undergraduate nursing | * Professional identity formation: Present — end-of-life care as identity-forming professional experience. * Ethical reasoning: Present — ethical dimensions of end-of-life care central. * Moral agency: Present — moral distress and agency in end-of-life contexts. * Clinical judgment: Present — clinical decision-making in palliative/end-of-life scenarios. * Digital professionalism: Not addressed. * Power awareness: Not addressed. * Recognition of clinical AI infrastructures: Not addressed. | Exploratory study using AI simulations for end-of-life education in undergraduate nursing. Finds AI-based simulations provide a safe space for students to practice difficult conversations and develop ethical reasoning, moral agency, and clinical judgment in palliative care contexts. |
| 39. | Brendan Martin & Michaela Reid | 2025 | Prevalence of artificial intelligence use and instruction in nursing education: A national study of prelicensure nursing programs in the United States | Empirical study (national survey/descriptive) | Multiple AI applications; prevalence of AI use and instruction in US prelicensure nursing programs | * Professional identity formation: Not addressed. * Ethical reasoning: Not addressed. * Moral agency: Not addressed. * Clinical judgment: Not addressed. * Digital professionalism: Present — AI instruction as digital competency development. * Power awareness: Not addressed. * Recognition of clinical AI infrastructures: Not addressed. | National survey of US prelicensure nursing programs on prevalence of AI use and formal AI instruction. Finds AI tools widely used by students but formal AI instruction rare. Significant variability in institutional approaches; majority of programs lack explicit AI policies or curricula. |
| 40. | DW La Rosa & JA Rossetti | N/A | Integrating AI into the Nursing Curriculum | Commentary/editorial | Multiple AI applications; integrating AI into the nursing curriculum | * Professional identity formation: Not addressed. * Ethical reasoning: Not addressed. * Moral agency: Not addressed. * Clinical judgment: Not addressed. * Digital professionalism: Present — AI as a core curricular component. * Power awareness: Not addressed. * Recognition of clinical AI infrastructures: Not addressed. | Discusses frameworks and strategies for integrating AI into the nursing curriculum. Advocates for proactive curricular redesign that embeds AI competencies across all levels of nursing education, with attention to both technical skills and critical evaluation of AI tools. |
| 41. | Silva & Sheets | 2025 | Reimagining Graduate Study With Generative Artificial Intelligence-Supported Study Strategies: A Doctor of Nursing Practice Student’s Perspective. | Commentary/editorial | ChatGPT/generative AI; AI-supported study strategies in Doctor of Nursing Practice education | * Professional identity formation: Not addressed. * Ethical reasoning: Not addressed. * Moral agency: Not addressed. * Clinical judgment: Not addressed. * Digital professionalism: Present — responsible use of AI study tools. * Power awareness: Not addressed. * Recognition of clinical AI infrastructures: Not addressed. | Student perspective on reimagining graduate study using generative AI-supported strategies in a DNP program. Describes practical applications of AI for literature synthesis, writing support, and study planning, while noting the importance of maintaining academic integrity and critical engagement. |
| 42. | Hamilton et al. | 2026 | Application of generative artificial intelligence by nurse academics in higher education: A scoping review. | Scoping review | Multiple AI applications; generative AI use by nurse academics in higher education | * Professional identity formation: Not addressed. * Ethical reasoning: Present — ethical concerns about AI in academic practice. * Moral agency: Not addressed. * Clinical judgment: Not addressed. * Digital professionalism: Present — digital competency of nurse academics. * Power awareness: Not addressed. * Recognition of clinical AI infrastructures: Not addressed. | Scoping review of generative AI applications by nurse academics in higher education. Identifies uses for curriculum development, assessment design, and scholarly writing. Highlights ethical concerns about authorship, academic integrity, and the need for institutional AI governance frameworks. |
| 43. | Sharon K. Titus et al. | 2025 | Academic Nurse Educators’ Ethical Concerns About the Use of GenAI in Nursing Education | Empirical study (qualitative/survey) | ChatGPT/generative AI; academic nurse educators’ ethical concerns about GenAI in nursing education | * Professional identity formation: Not addressed. * Ethical reasoning: Present — central focus on ethical concerns of nurse educators. * Moral agency: Present — educator moral responsibility in AI governance. * Clinical judgment: Not addressed. * Digital professionalism: Present — responsible AI use in academic practice. * Power awareness: Not addressed. * Recognition of clinical AI infrastructures: Not addressed. | Qualitative study of academic nurse educators’ ethical concerns about generative AI. Key concerns include academic dishonesty, erosion of critical thinking, lack of institutional guidance, and uncertainty about appropriate boundaries for AI use in assessment and clinical education. |
| 44. | Fang et al. | 2025 | Publishing Policies on AI-Generated Content in Nursing: An Evidence Base for Shaping Educational Policy and Upholding Academic Integrity. | Literature review/policy analysis | ChatGPT/generative AI; publishing policies on AI-generated content in nursing | * Professional identity formation: Not addressed. * Ethical reasoning: Present — academic integrity and publication ethics. * Moral agency: Present — authorship responsibility and moral agency. * Clinical judgment: Not addressed. * Digital professionalism: Present — professional standards for AI use in scholarly publishing. * Power awareness: Not addressed. * Recognition of clinical AI infrastructures: Not addressed. | Analyses publishing policies on AI-generated content across nursing journals. Finds highly variable and often unclear policies; argues for standardised guidelines that uphold academic integrity while enabling responsible use of AI writing tools. Provides evidence base for shaping educational and publishing policy. |
| 45. | Jessica Grimm et al. | 2025 | Addressing Generative AI Detection in Doctoral Nursing Education | Commentary/editorial | ChatGPT/generative AI; AI detection in doctoral nursing education | * Professional identity formation: Not addressed. * Ethical reasoning: Present — academic integrity and ethical AI use. * Moral agency: Present — doctoral student moral responsibility. * Clinical judgment: Not addressed. * Digital professionalism: Present — responsible AI use in doctoral education. * Power awareness: Not addressed. * Recognition of clinical AI infrastructures: Not addressed. | Addresses the challenge of detecting generative AI use in doctoral nursing education. Argues that detection-focused approaches are insufficient; advocates for assessment redesign and explicit teaching of ethical AI use as a professional competency for doctoral nurse leaders. |
| 46. | Mary Estelle Bester & Kathryn Zeigler | 2025 | Teaching Nursing Students Effective Artificial Intelligence Prompt Engineering | Commentary/editorial | ChatGPT/generative AI; prompt engineering education for nursing students | * Professional identity formation: Not addressed. * Ethical reasoning: Not addressed. * Moral agency: Not addressed. * Clinical judgment: Not addressed. * Digital professionalism: Present — prompt engineering as a professional AI skill. * Power awareness: Not addressed. * Recognition of clinical AI infrastructures: Not addressed. | Describes an approach to teaching nursing students effective AI prompt engineering. Argues that prompt engineering is an essential emerging competency for nursing practice and education. Provides practical frameworks for teaching students to interact effectively and critically with AI tools. |
| 47. | Dan Song et al. | 2025 | Effects of generative artificial intelligence on higher-order thinking skills and artificial intelligence literacy in nursing undergraduates: A quasi-experimental study | Empirical study (quasi-experimental) | ChatGPT/generative AI; effects on higher-order thinking skills and AI literacy in nursing undergraduates | * Professional identity formation: Not addressed. * Ethical reasoning: Not addressed. * Moral agency: Not addressed. * Clinical judgment: Present — higher-order thinking linked to clinical reasoning. * Digital professionalism: Present — AI literacy as outcome measure. * Power awareness: Not addressed. * Recognition of clinical AI infrastructures: Not addressed. | Quasi-experimental study examining effects of generative AI use on higher-order thinking skills and AI literacy in undergraduate nursing students. Finds significant improvements in AI literacy and some higher-order thinking skills in the AI-integrated group, with implications for pedagogical design. |
| 48. | Tracy M. Dodson et al. | 2025 | Nursing students’ AI literacy and ethical understanding of AI in nursing education | Empirical study (survey/descriptive) | Multiple AI applications; nursing students’ AI literacy and ethical understanding of AI in nursing education | * Professional identity formation: Not addressed. * Ethical reasoning: Present — ethical understanding of AI as a key outcome. * Moral agency: Not addressed. * Clinical judgment: Not addressed. * Digital professionalism: Present — AI literacy as core competency. * Power awareness: Not addressed. * Recognition of clinical AI infrastructures: Not addressed. | Surveys nursing students’ AI literacy levels and ethical understanding of AI in nursing education. Finds variable AI literacy with gaps in ethical knowledge; students demonstrate awareness of AI capabilities but limited understanding of algorithmic bias, data privacy, and professional accountability implications. |
| 49. | Ragi George et al. | 2025 | Nurse Educators’ Concerns of GenAI in Education: Scoping Review of Technical Factors | Scoping review | ChatGPT/generative AI; nurse educators’ technical concerns about GenAI in education | * Professional identity formation: Not addressed. * Ethical reasoning: Present — ethical dimensions of AI in education. * Moral agency: Not addressed. * Clinical judgment: Not addressed. * Digital professionalism: Present — technical competency for AI use. * Power awareness: Not addressed. * Recognition of clinical AI infrastructures: Not addressed. | Scoping review of nurse educators’ technical concerns about generative AI in education. Identifies concerns about accuracy and hallucination in AI outputs, data privacy, algorithmic bias, and lack of technical expertise among nursing faculty. Calls for technical training and institutional support structures. |
| 50. | Y Li et al. | N/A | Nursing Students’ Perceptions and Attitudes on the Application of Artificial Intelligence in Nursing Education: A Mixed‐Methods Systematic Review | Systematic review (mixed methods) | Multiple AI applications; nursing students’ perceptions and attitudes on AI in nursing education | * Professional identity formation: Not addressed. * Ethical reasoning: Present — ethical attitudes toward AI surveyed. * Moral agency: Not addressed. * Clinical judgment: Not addressed. * Digital professionalism: Present — AI competency and readiness. * Power awareness: Not addressed. * Recognition of clinical AI infrastructures: Not addressed. | Mixed-methods systematic review of nursing students’ perceptions and attitudes toward AI in nursing education. Finds generally positive attitudes with openness to AI integration, but concerns about job displacement, ethical risks, and lack of preparedness. Highlights need for structured AI education. |
| 51. | Mohammad Faisal Alali | 2025 | The impact of artificial intelligence on nursing students: A systematic review | Systematic review | Multiple AI applications; impact of AI on nursing students broadly | * Professional identity formation: Not addressed. * Ethical reasoning: Present — ethical implications of AI for nursing students. * Moral agency: Not addressed. * Clinical judgment: Present — AI impact on clinical reasoning. * Digital professionalism: Present — digital competency development. * Power awareness: Not addressed. * Recognition of clinical AI infrastructures: Not addressed. | Systematic review of the impact of AI on nursing students across educational and clinical domains. Identifies positive impacts on learning efficiency and clinical skill development, alongside risks of over-reliance, reduced critical thinking, and ethical uncertainties. Calls for balanced integration strategies. |
| 52. | Tuba Şengül et al. | 2025 | Ethical decision-making and artificial intelligence in nursing education: An integrative review | Integrative review | AI ethics/bias; ethical decision-making and AI in nursing education | * Professional identity formation: Not addressed. * Ethical reasoning: Present — central focus of the review. * Moral agency: Present — moral decision-making in AI contexts. * Clinical judgment: Present — ethical clinical judgment. * Digital professionalism: Not addressed. * Power awareness: Not addressed. * Recognition of clinical AI infrastructures: Not addressed. | Integrative review of ethical decision-making and AI in nursing education. Finds that current nursing curricula inadequately prepare students for ethical dilemmas posed by AI in clinical practice. Calls for explicit integration of AI ethics content into nursing education frameworks. |
| 53. | Zeng et al. | No p | Developing a Nursing Research Education Agent Using Knowledge Graphs and Large Language Models: A Proof-of-Concept Study. | Empirical study (proof-of-concept) | Multiple AI applications; LLMs; knowledge graphs and LLMs for nursing research education | * Professional identity formation: Not addressed. * Ethical reasoning: Not addressed. * Moral agency: Not addressed. * Clinical judgment: Not addressed. * Digital professionalism: Present — advanced AI tool use for research education. * Power awareness: Not addressed. * Recognition of clinical AI infrastructures: Present — AI infrastructure for research support. | Proof-of-concept study developing a nursing research education agent using knowledge graphs and large language models. Demonstrates feasibility of AI-assisted research mentoring, with the agent providing structured guidance on research methodology, literature synthesis, and study design. |
| 54. | Gokalp et al. | 2025 | Readability, reliability, and quality of nursing care plan texts generated by ChatGPT. | Empirical study (descriptive/analytical) | ChatGPT/generative AI; readability, reliability, and quality of ChatGPT-generated nursing care plans | * Professional identity formation: Not addressed. * Ethical reasoning: Not addressed. * Moral agency: Not addressed. * Clinical judgment: Present — quality of AI-generated clinical reasoning assessed. * Digital professionalism: Not addressed. * Power awareness: Not addressed. * Recognition of clinical AI infrastructures: Present — AI tools in clinical documentation. | Evaluates readability, reliability, and quality of nursing care plan texts generated by ChatGPT. Finds ChatGPT-generated care plans are readable and structurally sound but lack individualisation and clinical nuance. Raises concerns about uncritical adoption of AI-generated clinical documentation. |
| 55. | Canales Morales | 2025 | Ethics and Technology in Nursing Education: Rethinking Artificial Intelligence. | Commentary/editorial | AI ethics/bias; ethics and technology in nursing education, rethinking AI | * Professional identity formation: Not addressed. * Ethical reasoning: Present — central focus. * Moral agency: Present — moral responsibility in AI-integrated education. * Clinical judgment: Not addressed. * Digital professionalism: Present — ethical digital practice. * Power awareness: Present — power dynamics in technology adoption. * Recognition of clinical AI infrastructures: Not addressed. | Critical commentary on ethics and technology in nursing education. Argues for a fundamental rethinking of how AI is framed in nursing curricula, moving beyond technocentric efficiency narratives to foreground ethical responsibility, moral agency, and the social dimensions of AI in healthcare. |
| 56. | YO Marboah Kusi & L Nono Wete | N/A | Impact of Artificial Intelligence on Nursing Education | Literature review | Multiple AI applications; impact of AI on nursing education broadly | * Professional identity formation: Not available in metadata. * Ethical reasoning: Not available in metadata. * Moral agency: Not available in metadata. * Clinical judgment: Not available in metadata. * Digital professionalism: Not available in metadata. * Power awareness: Not available in metadata. * Recognition of clinical AI infrastructures: Not available in metadata. | Literature review of AI’s impact on nursing education. Limited metadata available for full extraction. Focus on broad impacts of AI technologies on nursing educational practices and outcomes. |
| 57. | Grace Sun | 2025 | Integrating artificial intelligence into nurse practitioner education: Strategies for teaching the next generation of nurse practitioners | Commentary/editorial | Multiple AI applications; integrating AI into nurse practitioner education | * Professional identity formation: Present — NP professional identity in AI era. * Ethical reasoning: Present — ethical AI use in advanced practice. * Moral agency: Not addressed. * Clinical judgment: Present — AI support for NP clinical reasoning. * Digital professionalism: Present — AI competency for NPs. * Power awareness: Not addressed. * Recognition of clinical AI infrastructures: Present — clinical AI tools for NPs. | Discusses strategies for integrating AI into nurse practitioner education to prepare the next generation of NPs. Emphasises the need for AI competency development, ethical frameworks, and clinical AI literacy as core components of advanced practice nursing education. |
| 58. | Moustaq Karim Khan Rony et al. | 2025 | Nursing educators’ perspectives on the integration of artificial intelligence into academic settings | Empirical study (qualitative/survey) | Multiple AI applications; nursing educators’ perspectives on AI integration in academic settings | * Professional identity formation: Not addressed. * Ethical reasoning: Present — ethical concerns about AI in academic settings. * Moral agency: Not addressed. * Clinical judgment: Not addressed. * Digital professionalism: Present — faculty AI competency. * Power awareness: Not addressed. * Recognition of clinical AI infrastructures: Not addressed. | Qualitative study of nursing educators’ perspectives on integrating AI into academic settings. Identifies opportunities (enhanced teaching, personalised learning) and challenges (ethical concerns, workload, lack of training). Calls for institutional support and faculty development in AI pedagogy. |
| 59. | Tuba Sengul et al. | 2025 | Evaluation of nursing students’ ethical decision-making biases and attitudes toward artificial intelligence in nursing education | Empirical study (survey/cross-sectional) | AI ethics/bias; nursing students’ ethical decision-making biases and attitudes toward AI in nursing education | * Professional identity formation: Not addressed. * Ethical reasoning: Present — ethical decision-making biases measured. * Moral agency: Present — moral reasoning in AI contexts assessed. * Clinical judgment: Present — clinical ethical judgment. * Digital professionalism: Not addressed. * Power awareness: Not addressed. * Recognition of clinical AI infrastructures: Not addressed. | Cross-sectional study evaluating nursing students’ ethical decision-making biases and attitudes toward AI in nursing education. Finds significant variability in ethical reasoning capacity; identifies cognitive biases that may affect ethical AI use. Recommends targeted ethics education integrated with AI literacy training. |
| 60. | Rahman | 2026 | Algorithmic bias and transparency in artificial intelligence tools for nursing education: A scoping review. | Scoping review | AI ethics/bias; algorithmic bias and transparency in AI tools for nursing education | * Professional identity formation: Not addressed. * Ethical reasoning: Present — bias and transparency as ethical issues. * Moral agency: Not addressed. * Clinical judgment: Not addressed. * Digital professionalism: Present — critical AI literacy. * Power awareness: Present — algorithmic power and transparency. * Recognition of clinical AI infrastructures: Present — AI tools in educational settings. | Scoping review of algorithmic bias and transparency in AI tools used in nursing education. Finds pervasive algorithmic bias in educational AI tools with limited transparency; argues for critical AI literacy as an essential nursing education competency to enable students to identify and challenge biased AI systems. |
| 61. | MI Rahman | N/A | Algorithmic Bias and Transparency in Artificial Intelligence Tools for Nursing Education: A Scoping Review | Scoping review | AI ethics/bias; algorithmic bias and transparency in AI tools for nursing education | * Professional identity formation: Not addressed. * Ethical reasoning: Present — bias and transparency. * Moral agency: Not addressed. * Clinical judgment: Not addressed. * Digital professionalism: Present — critical AI literacy. * Power awareness: Present — algorithmic power. * Recognition of clinical AI infrastructures: Present — AI tools in education. | Scoping review of algorithmic bias and transparency in AI tools for nursing education. (Note: appears to be same study as Paper 62/Rahman 2026 — possible duplicate or preprint/published version pair.) |
| 62. | K Mikkonen | N/A | The Evolution and Future of Nursing Education: Bridging Evidence-Based Practice and Hybrid Intelligence | Commentary/editorial | Multiple AI applications; evolution and future of nursing education with AI and evidence-based practice | * Professional identity formation: Not addressed. * Ethical reasoning: Not addressed. * Moral agency: Not addressed. * Clinical judgment: Present — evidence-based practice and clinical reasoning. * Digital professionalism: Present — hybrid intelligence and digital competency. * Power awareness: Not addressed. * Recognition of clinical AI infrastructures: Not addressed. | Discusses the evolution and future of nursing education at the intersection of evidence-based practice and AI (hybrid intelligence). Argues for a bridging framework that integrates AI tools with established EBP principles to enhance clinical reasoning and professional development. |
| 63. | Estel·la Ramírez-Baraldes et al. | 2025 | Artificial intelligence in nursing: New opportunities and challenges | Commentary/editorial | Multiple AI applications; new opportunities and challenges of AI in nursing broadly | * Professional identity formation: Present — professional role in AI-integrated nursing. * Ethical reasoning: Present — ethical challenges of AI in nursing. * Moral agency: Not addressed. * Clinical judgment: Present — AI and clinical decision-making. * Digital professionalism: Present — digital nursing competencies. * Power awareness: Not addressed. * Recognition of clinical AI infrastructures: Present — clinical AI systems. | Reviews new opportunities and challenges presented by AI in nursing across education and clinical practice. Argues for a proactive nursing response to AI integration that preserves the humanistic core of nursing while embracing technological advancement. |
| 64. | DM Alrazeeni et al. | N/A | Transforming Nursing Education with Artificial Intelligence: A Systematic Review (2010–2025) | Systematic review | Multiple AI applications; transforming nursing education with AI (2010-2025) | * Professional identity formation: Not addressed. * Ethical reasoning: Present — ethical AI integration. * Moral agency: Not addressed. * Clinical judgment: Present — AI and clinical skill development. * Digital professionalism: Present — AI competency in nursing education. * Power awareness: Not addressed. * Recognition of clinical AI infrastructures: Not addressed. | Systematic review (2010-2025) of AI transformation in nursing education. Maps the evolution from early simulation technologies to generative AI, identifying trends in AI adoption, evidence of educational effectiveness, and persistent gaps in faculty preparation and ethical frameworks. |
| 65. | Luciano Magalhães Vitorino et al. | 2025 | Artificial intelligence in nursing: advancing clinical judgment and decision-making | Commentary/editorial | Multiple AI applications; AI in nursing advancing clinical judgment and decision-making | * Professional identity formation: Not addressed. * Ethical reasoning: Not addressed. * Moral agency: Not addressed. * Clinical judgment: Present — central focus. * Digital professionalism: Present — AI competency for clinical nurses. * Power awareness: Not addressed. * Recognition of clinical AI infrastructures: Present — clinical AI decision support. | Discusses AI’s role in advancing clinical judgment and decision-making in nursing. Argues that AI tools can augment nursing clinical reasoning when integrated with strong foundational clinical education, but warns against substituting AI judgment for human clinical expertise. |
| 66. | MM El-Banna et al. | N/A | AI literacy and competency in nursing education: preparing students and faculty members for an AI-enabled future-a systematic review and meta-analysis | Systematic review and meta-analysis | Multiple AI applications; AI literacy and competency in nursing education for students and faculty | * Professional identity formation: Not addressed. * Ethical reasoning: Present — ethical AI competency. * Moral agency: Not addressed. * Clinical judgment: Not addressed. * Digital professionalism: Present — AI literacy and competency as core outcomes. * Power awareness: Not addressed. * Recognition of clinical AI infrastructures: Not addressed. | Systematic review and meta-analysis of AI literacy and competency in nursing education. Finds significant deficits in AI literacy among both students and faculty; identifies key competency domains; provides evidence base for developing AI literacy frameworks for nursing education programs. |
| 67. | Mirko Prosen & Sabina Ličen | 2025 | Perceptions, Ethical Challenges and Sustainable Integration of Generative AI in Health Science Education: A Cross-Sectional Study | Empirical study (cross-sectional survey) | ChatGPT/generative AI; perceptions, ethical challenges, and sustainable integration of generative AI in health science education | * Professional identity formation: Not addressed. * Ethical reasoning: Present — ethical challenges of generative AI. * Moral agency: Not addressed. * Clinical judgment: Not addressed. * Digital professionalism: Present — sustainable AI integration. * Power awareness: Not addressed. * Recognition of clinical AI infrastructures: Not addressed. | Cross-sectional study of health science students and educators on perceptions, ethical challenges, and sustainable integration of generative AI. Finds positive perceptions tempered by ethical concerns about AI reliability. Proposes a sustainability framework for responsible AI integration in health science education. |
| 68. | Sara Shishehgar et al. | 2025 | Artificial intelligence in health education and practice: A systematic review of health students’ and academics’ knowledge, perceptions and experiences | Systematic review | Multiple AI applications; health students’ and academics’ knowledge, perceptions, and experiences of AI in health education and practice | * Professional identity formation: Not addressed. * Ethical reasoning: Present — ethical dimensions of AI in health education. * Moral agency: Not addressed. * Clinical judgment: Present — AI and clinical reasoning. * Digital professionalism: Present — AI knowledge and competency. * Power awareness: Not addressed. * Recognition of clinical AI infrastructures: Present — AI in clinical practice settings. | Systematic review of health students’ and academics’ knowledge, perceptions, and experiences of AI. Finds knowledge gaps and variable perceptions across health professions; identifies need for standardised AI education frameworks across health professions education. |
| 69. | El-Ashry et al. | 2026 | Attitudes toward artificial intelligence and impostor phenomenon among nursing students: A five-country cross-sectional study. | Empirical study (cross-sectional survey, multi-country) | Multiple AI applications; attitudes toward AI and impostor phenomenon among nursing students | * Professional identity formation: Present — impostor phenomenon linked to professional identity. * Ethical reasoning: Not addressed. * Moral agency: Not addressed. * Clinical judgment: Not addressed. * Digital professionalism: Present — AI attitudes and competency confidence. * Power awareness: Present — power dynamics in AI-professional identity relationship. * Recognition of clinical AI infrastructures: Not addressed. | Five-country cross-sectional study of nursing students’ attitudes toward AI and impostor phenomenon. Finds significant associations between AI anxiety, impostor syndrome, and professional identity uncertainty. Highlights psychological dimensions of AI integration on nursing student professional development. |
| 70. | Jones et al. | 2025 | Enhancing the Objective Structured Clinical Examination Using Artificial Intelligence. | Empirical study (descriptive/development study) | AI in simulation/education; AI enhancement of OSCE (Objective Structured Clinical Examination) | * Professional identity formation: Not addressed. * Ethical reasoning: Not addressed. * Moral agency: Not addressed. * Clinical judgment: Present — OSCE as clinical judgment assessment. * Digital professionalism: Not addressed. * Power awareness: Not addressed. * Recognition of clinical AI infrastructures: Present — AI in clinical assessment infrastructure. | Describes and evaluates AI enhancement of the Objective Structured Clinical Examination (OSCE). Finds AI can improve standardisation, feedback quality, and efficiency of OSCE assessment. Raises questions about the role of human judgment in clinical competency assessment when AI is involved. |
| 71. | Animesh Ghimire | 2025 | Redefining pedagogy with artificial intelligence: How nursing students are shaping the future of learning | Commentary/editorial | Multiple AI applications; redefining pedagogy with AI and nursing students’ role in future learning | * Professional identity formation: Present — nursing students as active shapers of AI-integrated futures. * Ethical reasoning: Not addressed. * Moral agency: Not addressed. * Clinical judgment: Not addressed. * Digital professionalism: Present — AI-transformed pedagogy. * Power awareness: Not addressed. * Recognition of clinical AI infrastructures: Not addressed. | Argues that AI is fundamentally redefining nursing pedagogy, with nursing students positioned as active shapers of AI-integrated learning futures. Calls for student-centred approaches to AI integration that build agency, critical thinking, and professional identity in the AI era. |
| 72. | Lai et al. | 2026 | Artificial intelligence literacy in higher education and implications for nursing education: A scoping review. | Scoping review | Multiple AI applications; AI literacy in higher education and implications for nursing education | * Professional identity formation: Not addressed. * Ethical reasoning: Present — ethical AI literacy. * Moral agency: Not addressed. * Clinical judgment: Not addressed. * Digital professionalism: Present — AI literacy as core competency. * Power awareness: Not addressed. * Recognition of clinical AI infrastructures: Not addressed. | Scoping review of AI literacy in higher education with specific implications for nursing education. Identifies key components of AI literacy (technical, ethical, critical), maps current evidence on AI literacy education, and proposes a framework for embedding AI literacy across nursing curricula. |
| 73. | Gianluca Mondillo et al. | 2025 | The Role of Generative Artificial Intelligence in Pediatric Healthcare Professions Education: A Narrative Review | Narrative review | ChatGPT/generative AI; role of generative AI in pediatric healthcare professions education | * Professional identity formation: Not addressed. * Ethical reasoning: Present — ethical use of AI in healthcare education. * Moral agency: Not addressed. * Clinical judgment: Present — AI support for clinical reasoning in pediatrics. * Digital professionalism: Present — responsible AI use in health professions education. * Power awareness: Not addressed. * Recognition of clinical AI infrastructures: Not addressed. | Narrative review of generative AI’s role in pediatric healthcare professions education. Identifies applications in case-based learning, clinical scenario generation, and formative assessment. Notes ethical concerns specific to pediatric contexts and calls for age-appropriate, evidence-based AI integration guidelines. |
| 74. | Ida Ayu Agung Laksmi et al. | 2025 | Utilización de la Inteligencia Artificial en la educación de Enfermería: una revisión del alcance | Scoping review | Multiple AI applications; AI use in nursing education (Spanish-language scoping review) | * Professional identity formation: Not addressed. * Ethical reasoning: Not addressed. * Moral agency: Not addressed. * Clinical judgment: Present — AI and clinical skill development. * Digital professionalism: Present — digital competency in nursing education. * Power awareness: Not addressed. * Recognition of clinical AI infrastructures: Not addressed. | Spanish-language scoping review of AI use in nursing education. Maps AI applications across simulation, clinical training, and didactic instruction. Identifies gaps in Spanish-language nursing education research and calls for culturally contextualised AI integration frameworks. |
| 75. | Erol et al. | 2026 | Perceived professional competence and workplace violence management ability among nursing students: a cross-sectional study. | Empirical study (cross-sectional survey) | AI in simulation/education; perceived professional competence and workplace violence management among nursing students | * Professional identity formation: Present — professional competence and identity. * Ethical reasoning: Not addressed. * Moral agency: Not addressed. * Clinical judgment: Present — professional competence assessment. * Digital professionalism: Not addressed. * Power awareness: Present — workplace violence as power dynamic. * Recognition of clinical AI infrastructures: Not addressed. | Cross-sectional study of perceived professional competence and workplace violence management ability among nursing students. While not primarily about AI, examines professional formation outcomes relevant to AI-integrated clinical education contexts. |
| 76. | Akter et al. | 2025 | Nurses’ perspectives on barriers to artificial intelligence integration in clinical practice: a qualitative phenomenological study. | Empirical study (qualitative phenomenological) | Multiple AI applications; nurses’ perspectives on barriers to AI integration in clinical practice | * Professional identity formation: Present — nursing professional identity in AI-integrated practice. * Ethical reasoning: Present — ethical barriers to AI. * Moral agency: Present — nurse agency in AI adoption decisions. * Clinical judgment: Present — AI and clinical decision-making. * Digital professionalism: Present — digital competency barriers. * Power awareness: Present — institutional power in AI adoption. * Recognition of clinical AI infrastructures: Present — clinical AI systems as barrier context. | Qualitative phenomenological study of nurses’ perspectives on barriers to AI integration in clinical practice. Identifies key barriers: lack of training, concerns about patient safety, ethical uncertainties, workload implications, and institutional power dynamics. Highlights the importance of nurse agency in AI adoption processes. |
| 77. | Nirgiz et al. | 2026 | The relationship between nurses’ anxiety and attitudes towards artificial intelligence and examination of influencing factors. | Empirical study (cross-sectional survey) | Multiple AI applications; nurses’ anxiety and attitudes toward AI | * Professional identity formation: Not addressed. * Ethical reasoning: Not addressed. * Moral agency: Not addressed. * Clinical judgment: Not addressed. * Digital professionalism: Present — AI attitudes and readiness. * Power awareness: Not addressed. * Recognition of clinical AI infrastructures: Not addressed. | Cross-sectional study of nurses’ anxiety and attitudes toward AI and influencing factors. Finds significant AI anxiety among nurses, influenced by age, education level, and prior AI experience. Identifies need for targeted AI education and anxiety-reduction interventions in nursing workforce development. |
| 78. | Suen et al. | 2026 | Application of Artificial Intelligence in Nursing: A Bibliometric Analysis of Global Research Trends. | Empirical study (bibliometric analysis) | Multiple AI applications; global research trends in AI applications in nursing | * Professional identity formation: Not addressed. * Ethical reasoning: Not addressed. * Moral agency: Not addressed. * Clinical judgment: Not addressed. * Digital professionalism: Not addressed. * Power awareness: Not addressed. * Recognition of clinical AI infrastructures: Not addressed. | Bibliometric analysis of global research trends in AI applications in nursing. Maps publication growth, key themes, leading countries, and influential journals. Identifies clinical decision support and AI in nursing education as dominant research themes, with rapid acceleration post-2020. |
| 79. | Gürdap & Öner | 2026 | Nurses’ attitudes toward artificial intelligence applications and their clinical decision-making competence: A cross-sectional study. | Empirical study (cross-sectional survey) | Multiple AI applications; nurses’ attitudes toward AI and clinical decision-making competence | * Professional identity formation: Not addressed. * Ethical reasoning: Not addressed. * Moral agency: Not addressed. * Clinical judgment: Present — clinical decision-making competence as outcome. * Digital professionalism: Present — AI attitudes and competency. * Power awareness: Not addressed. * Recognition of clinical AI infrastructures: Not addressed. | Cross-sectional study of nurses’ attitudes toward AI applications and clinical decision-making competence. Finds positive correlations between positive AI attitudes and clinical decision-making confidence. Highlights the role of AI education in building both technical competency and professional confidence. |
| 80. | Aydogan et al. | 2026 | Artificial Intelligence Experiences of Nurse Educators: A Phenomenological Study. | Empirical study (qualitative phenomenological) | Multiple AI applications; nurse educators’ AI experiences | * Professional identity formation: Present — nurse educator identity in AI era. * Ethical reasoning: Present — ethical dimensions of AI use in education. * Moral agency: Present — educator agency in AI adoption. * Clinical judgment: Not addressed. * Digital professionalism: Present — AI competency development of nurse educators. * Power awareness: Not addressed. * Recognition of clinical AI infrastructures: Not addressed. | Phenomenological study of nurse educators’ lived experiences with AI. Identifies themes of uncertainty, adaptation, and professional identity renegotiation as educators navigate AI integration. Highlights the need for institutional support and professional development for nurse educators in the AI era. |
| 81. | Thuy Minh Ha & Zarrin Seema Siddiqui | 2025 | Understanding medical students’ transition to clinical training: a qualitative study of transformative learning and professional identity formation | Empirical study (qualitative) | AI in simulation/education; medical students’ transition to clinical training and professional identity formation | * Professional identity formation: Present — central focus of the study. * Ethical reasoning: Present — ethical dimensions of clinical training. * Moral agency: Present — moral development in clinical transition. * Clinical judgment: Present — clinical reasoning development. * Digital professionalism: Not addressed. * Power awareness: Present — power dynamics in clinical learning environments. * Recognition of clinical AI infrastructures: Not addressed. | Qualitative study of medical students’ transition to clinical training using transformative learning theory. While focused on medical students, provides highly relevant frameworks for understanding professional identity formation in clinical AI contexts, including moral development and power dynamics. |
| 82. | Šimunić et al. | 2026 | Development of Digital Literacy: Application of Artificial Intelligence in Education and Clinical Practice of Oncology Nurses. | Empirical study (descriptive/mixed methods) | Multiple AI applications; AI in education and clinical practice of oncology nurses — digital literacy development | * Professional identity formation: Present — oncology nurse professional identity. * Ethical reasoning: Not addressed. * Moral agency: Not addressed. * Clinical judgment: Present — AI and clinical practice. * Digital professionalism: Present — digital literacy development. * Power awareness: Not addressed. * Recognition of clinical AI infrastructures: Present — AI in oncology clinical practice. | Describes development of digital literacy through AI applications in oncology nursing education and clinical practice. Finds AI tools support digital literacy development and clinical skill enhancement in oncology nurses, with implications for professional development frameworks. |
| 83. | Cheng | 2026 | [Digital Empowerment: Reshaping the Future of Nursing Care and Education]. | Commentary/editorial | Multiple AI applications; digital empowerment and reshaping nursing care and education | * Professional identity formation: Not addressed. * Ethical reasoning: Not addressed. * Moral agency: Not addressed. * Clinical judgment: Present — AI reshaping clinical care. * Digital professionalism: Present — digital empowerment in nursing. * Power awareness: Not addressed. * Recognition of clinical AI infrastructures: Present — digital nursing infrastructure. | Commentary on digital empowerment and the reshaping of nursing care and education through AI. Argues for a vision of digitally empowered nursing that leverages AI to enhance care quality while preserving the relational and humanistic dimensions of nursing practice. |
| 84. | Rabie Adel El Arab et al. | 2025 | Integrative review of artificial intelligence applications in nursing: education, clinical practice, workload management, and professional perceptions | Integrative review | Multiple AI applications; AI applications in nursing across education, clinical practice, workload management, and professional perceptions | * Professional identity formation: Present — professional perceptions of AI. * Ethical reasoning: Present — ethical dimensions of AI in nursing. * Moral agency: Not addressed. * Clinical judgment: Present — AI in clinical decision-making. * Digital professionalism: Present — AI competency across nursing domains. * Power awareness: Not addressed. * Recognition of clinical AI infrastructures: Present — clinical AI systems. | Integrative review of AI applications across nursing education, clinical practice, workload management, and professional perceptions. Provides comprehensive mapping of AI’s multidimensional impact on nursing, identifying opportunities for AI-enhanced practice and persistent gaps in ethical frameworks and workforce preparation. |
| 85. | Velibor Božić | 2024 | Artifical Intelligence in nurse education | Literature review | Multiple AI applications; AI in nurse education broadly | * Professional identity formation: Not addressed. * Ethical reasoning: Not addressed. * Moral agency: Not addressed. * Clinical judgment: Not addressed. * Digital professionalism: Present — AI in nursing education. * Power awareness: Not addressed. * Recognition of clinical AI infrastructures: Not addressed. | Literature review of artificial intelligence in nurse education. Reviews the landscape of AI applications in nursing education contexts, identifying key developments and implications for nursing educational practice. |
| 86. | Kohanová et al. | 2026 | Perceived patient safety competencies among Slovak nursing students: a psychometric analysis. | Empirical study (psychometric/cross-sectional) | AI in simulation/education; patient safety competencies among nursing students | * Professional identity formation: Present — patient safety competence as professional formation. * Ethical reasoning: Present — safety and ethical practice. * Moral agency: Not addressed. * Clinical judgment: Present — patient safety clinical judgment. * Digital professionalism: Not addressed. * Power awareness: Not addressed. * Recognition of clinical AI infrastructures: Not addressed. | Psychometric analysis of perceived patient safety competencies among Slovak nursing students. While not primarily about AI, addresses professional formation outcomes (patient safety competence) relevant to AI-integrated nursing education contexts. |
| 87. | SY Yu et al. | N/A | Artificial intelligence-enhanced clinical reasoning in nurse practitioners: A systematic review | Systematic review | Multiple AI applications; AI-enhanced clinical reasoning in nurse practitioners | * Professional identity formation: Present — NP professional identity and AI. * Ethical reasoning: Not addressed. * Moral agency: Not addressed. * Clinical judgment: Present — AI-enhanced clinical reasoning as central focus. * Digital professionalism: Present — AI competency for NPs. * Power awareness: Not addressed. * Recognition of clinical AI infrastructures: Present — AI clinical tools for NPs. | Systematic review of AI-enhanced clinical reasoning in nurse practitioners. Finds emerging evidence that AI decision support tools can augment NP clinical reasoning, but highlights need for critical evaluation skills and cautions against over-reliance on AI recommendations in advanced practice. |
| 88. | Abdi et al. | 2026 | Predicting the adoption of evidence-based practice (EBP) based on critical thinking levels among nursing students. | Empirical study (cross-sectional) | AI in simulation/education; predicting evidence-based practice adoption based on critical thinking among nursing students | * Professional identity formation: Not addressed. * Ethical reasoning: Not addressed. * Moral agency: Not addressed. * Clinical judgment: Present — critical thinking and EBP adoption. * Digital professionalism: Not addressed. * Power awareness: Not addressed. * Recognition of clinical AI infrastructures: Not addressed. | Cross-sectional study predicting EBP adoption based on critical thinking levels among nursing students. While not primarily about AI, examines critical thinking as a predictor of EBP adoption, relevant to AI-integrated clinical education and the development of clinical judgment. |
| 89. | Prof. Annal Sathiya | 2025 | AI In Nursing Education: Transforming Pedagogy for the Future Nursing | Commentary/editorial | Multiple AI applications; AI in nursing education transforming pedagogy for the future | * Professional identity formation: Not addressed. * Ethical reasoning: Not addressed. * Moral agency: Not addressed. * Clinical judgment: Not addressed. * Digital professionalism: Present — AI-transformed pedagogy. * Power awareness: Not addressed. * Recognition of clinical AI infrastructures: Not addressed. | Commentary on AI in nursing education and its transformative potential for nursing pedagogy. Argues for proactive curricular redesign that positions AI as a pedagogical tool for enhancing learning outcomes and preparing future nurses for AI-integrated healthcare environments. |
| 90. | Gouifrane et al. | 2026 | Effect of flipped classroom and screen-based simulation on nursing students’ clinical judgment: A quasi-experimental study. | Empirical study (quasi-experimental) | AI in simulation/education; flipped classroom and screen-based simulation on nursing students’ clinical judgment | * Professional identity formation: Not addressed. * Ethical reasoning: Not addressed. * Moral agency: Not addressed. * Clinical judgment: Present — central outcome measure. * Digital professionalism: Not addressed. * Power awareness: Not addressed. * Recognition of clinical AI infrastructures: Not addressed. | Quasi-experimental study of flipped classroom combined with screen-based simulation on nursing students’ clinical judgment. Finds significant improvements in clinical judgment in the intervention group, with implications for technology-enhanced nursing education design. |
| 91. | Leigh Montejo et al. | 2024 | Artificial intelligence (AI) applications in healthcare and considerations for nursing education | Literature review | Multiple AI applications; AI applications in healthcare and considerations for nursing education | * Professional identity formation: Not addressed. * Ethical reasoning: Present — ethical considerations of AI in healthcare. * Moral agency: Not addressed. * Clinical judgment: Present — AI and clinical decision-making. * Digital professionalism: Present — AI competency for nurses. * Power awareness: Not addressed. * Recognition of clinical AI infrastructures: Present — AI in healthcare systems. | Reviews AI applications in healthcare with specific considerations for nursing education. Identifies key AI technologies transforming healthcare and argues for nursing education to proactively prepare graduates for AI-integrated clinical environments through targeted competency development. |
| 92. | P Canales-Morales | N/A | Ethics and Technology in Nursing Education: Rethinking Artificial Intelligence | Commentary/editorial | AI ethics/bias; ethics and technology in nursing education, rethinking AI | * Professional identity formation: Not addressed. * Ethical reasoning: Present — central focus. * Moral agency: Present — moral responsibility in AI-integrated education. * Clinical judgment: Not addressed. * Digital professionalism: Present — ethical digital practice. * Power awareness: Present — power dynamics in technology adoption. * Recognition of clinical AI infrastructures: Not addressed. | Critical commentary on ethics and technology in nursing education. (Note: appears to be same study as Paper 56/Canales Morales 2025 — possible duplicate or preprint/published version pair.) |
| 93. | AL Watson et al. | N/A | Generative AI at the Bedside: An Integrative Review of Applications and Implications in Clinical Nursing Practice | Integrative review | ChatGPT/generative AI; generative AI applications and implications in clinical nursing practice | * Professional identity formation: Present — nursing professional identity in AI-integrated practice. * Ethical reasoning: Present — ethical implications of generative AI at bedside. * Moral agency: Present — nurse moral agency in AI-assisted care. * Clinical judgment: Present — AI and clinical decision-making at bedside. * Digital professionalism: Present — professional standards for AI use in clinical practice. * Power awareness: Present — power dynamics of AI in clinical settings. * Recognition of clinical AI infrastructures: Present — generative AI as clinical infrastructure. | Integrative review of generative AI applications and implications in clinical nursing practice. Identifies diverse applications (documentation, clinical decision support, patient communication) alongside significant concerns about accuracy, bias, accountability, and the displacement of nursing judgment. |
| 94. | E Nassef & IA Zeid | N/A | Artificial intelligence in nursing education: a descriptive literature review of the evidence | Literature review | Multiple AI applications; AI in nursing education — descriptive literature review | * Professional identity formation: Not addressed. * Ethical reasoning: Not addressed. * Moral agency: Not addressed. * Clinical judgment: Not addressed. * Digital professionalism: Present — AI in nursing education broadly. * Power awareness: Not addressed. * Recognition of clinical AI infrastructures: Not addressed. | Descriptive literature review of evidence on AI in nursing education. Provides a broad overview of AI applications in nursing educational contexts, identifying key themes and evidence gaps in the existing literature. |
| 95. | Paygozar et al. | 2025 | Artificial intelligence use as a key predictor of clinical performance in nursing students: a cross-sectional study from Iran. | Empirical study (cross-sectional) | Multiple AI applications; AI use as predictor of clinical performance in nursing students | * Professional identity formation: Not addressed. * Ethical reasoning: Not addressed. * Moral agency: Not addressed. * Clinical judgment: Present — clinical performance as outcome. * Digital professionalism: Present — AI use and clinical competency. * Power awareness: Not addressed. * Recognition of clinical AI infrastructures: Not addressed. | Cross-sectional study from Iran examining AI use as a predictor of clinical performance in nursing students. Finds that AI tool use is a significant predictor of clinical performance scores, suggesting AI integration may enhance clinical skill development when used appropriately. |
| 96. | Basil et al. | 2026 | A scoping review of the use of generative artificial intelligence tools in health profession education. | Scoping review | ChatGPT/generative AI; use of generative AI tools in health profession education broadly | * Professional identity formation: Not addressed. * Ethical reasoning: Present — ethical use of generative AI in education. * Moral agency: Not addressed. * Clinical judgment: Not addressed. * Digital professionalism: Present — AI literacy in health professions education. * Power awareness: Not addressed. * Recognition of clinical AI infrastructures: Not addressed. | Scoping review of generative AI tool use in health profession education. Maps applications across medicine, nursing, pharmacy, and allied health; identifies common themes of academic integrity concerns, faculty preparedness gaps, and need for evidence-based frameworks for generative AI in health professions education. |
| 97. | Tri Pham et al. | 2025 | The impact of generative AI on health professional education: a systematic review in the context of student learning | Systematic review | ChatGPT/generative AI; impact of generative AI on health professional education and student learning | * Professional identity formation: Not addressed. * Ethical reasoning: Present — ethical implications of generative AI for student learning. * Moral agency: Not addressed. * Clinical judgment: Present — AI impact on clinical reasoning development. * Digital professionalism: Present — AI literacy in health professions education. * Power awareness: Not addressed. * Recognition of clinical AI infrastructures: Not addressed. | Systematic review of generative AI’s impact on health professional education in the context of student learning. Finds mixed evidence on learning outcomes; identifies concerns about critical thinking development and academic integrity alongside opportunities for personalised learning and feedback. |
| 98. | Laura Creighton et al. | 2025 | Promoting self-efficacy of nursing students in academic integrity through a digital serious game: a pre/post-test study | Empirical study (pre/post-test) | AI in simulation/education; digital serious game for academic integrity in nursing education | * Professional identity formation: Not addressed. * Ethical reasoning: Present — academic integrity as ethical competency. * Moral agency: Present — student moral agency in academic integrity. * Clinical judgment: Not addressed. * Digital professionalism: Present — digital learning tools for professional competency. * Power awareness: Not addressed. * Recognition of clinical AI infrastructures: Not addressed. | Pre/post-test study of a digital serious game promoting nursing students’ self-efficacy in academic integrity. Finds significant improvements in academic integrity self-efficacy, with implications for AI-era nursing education where academic integrity challenges are heightened. |
| 99. | Graham et al. | 2026 | The use of digital gamification, extended reality, artificial intelligence, and integrated digital learning tools in palliative care education of undergraduate nurses: A systematic review. | Systematic review | Multiple AI applications; digital gamification, extended reality, AI, and integrated digital tools in palliative care nursing education | * Professional identity formation: Present — palliative care as identity-forming professional experience. * Ethical reasoning: Present — ethical dimensions of palliative care education. * Moral agency: Present — moral development in palliative care contexts. * Clinical judgment: Present — clinical judgment in palliative care. * Digital professionalism: Present — digital tools in nursing education. * Power awareness: Not addressed. * Recognition of clinical AI infrastructures: Not addressed. | Systematic review of digital gamification, extended reality, AI, and integrated digital learning tools in palliative care nursing education. Finds evidence supporting digital tool effectiveness for palliative care education, with AI simulations particularly useful for difficult conversation practice and ethical reasoning development. |
| 100. | Elizabeth A. Johnson & Benjamin J. Galatzan | 2025 | A critical juncture: Reimagining nursing professional identity and regulation in the ethical integration of innovation and technology in healthcare | Commentary/editorial | Multiple AI applications; reimagining nursing professional identity and regulation in ethical AI integration | * Professional identity formation: Present — central focus; nursing professional identity at critical juncture. * Ethical reasoning: Present — ethical integration of innovation and technology. * Moral agency: Present — nursing moral agency in AI regulation. * Clinical judgment: Present — AI and nursing clinical practice. * Digital professionalism: Present — professional standards for AI in nursing. * Power awareness: Present — regulatory power and professional governance. * Recognition of clinical AI infrastructures: Present — healthcare AI systems and nursing regulation. | Critical commentary on nursing professional identity and regulation at the juncture of AI integration. Argues that nursing must proactively reimagine professional identity frameworks and regulatory standards to ensure ethical AI integration that preserves nursing’s humanistic core and professional accountability. |
| 101. | N Nacer et al. | N/A | Artificial Intelligence and Nursing Education: Enhancing Teaching, Learning, and Critical Thinking | Literature review | Multiple AI applications; AI and nursing education enhancing teaching, learning, and critical thinking | * Professional identity formation: Not addressed. * Ethical reasoning: Not addressed. * Moral agency: Not addressed. * Clinical judgment: Present — critical thinking and clinical reasoning. * Digital professionalism: Present — AI in nursing education. * Power awareness: Not addressed. * Recognition of clinical AI infrastructures: Not addressed. | Literature review of AI in nursing education with focus on enhancing teaching, learning, and critical thinking. Reviews evidence on AI tools that support critical thinking development, identifying promising approaches and calling for more rigorous evaluation of critical thinking outcomes. |
| 102. | RA El Arab et al. | N/A | The role of AI in nursing education and practice: umbrella review | Systematic review (umbrella review) | Multiple AI applications; role of AI in nursing education and practice — umbrella review | * Professional identity formation: Present — nursing professional identity in AI era. * Ethical reasoning: Present — ethical AI in nursing. * Moral agency: Not addressed. * Clinical judgment: Present — AI and clinical practice. * Digital professionalism: Present — AI competency in nursing. * Power awareness: Not addressed. * Recognition of clinical AI infrastructures: Present — AI in clinical nursing practice. | Umbrella review synthesising evidence on AI’s role in nursing education and practice. Provides high-level synthesis of systematic review evidence, identifying consistent themes of AI potential for enhancing nursing education and practice alongside persistent gaps in ethical frameworks and workforce preparation. |
| 103. | Hallas & Spratling | 2026 | Guidance for Scholarly Writing: Assuring Ethical Publishing Standards for Nursing Professionals and Doctoral Students. | Commentary/editorial | Multiple AI applications; ethical publishing standards for nursing professionals and doctoral students | * Professional identity formation: Not addressed. * Ethical reasoning: Present — scholarly ethics and publishing standards. * Moral agency: Present — authorship responsibility and moral agency. * Clinical judgment: Not addressed. * Digital professionalism: Present — professional standards in AI-era publishing. * Power awareness: Not addressed. * Recognition of clinical AI infrastructures: Not addressed. | Guidance for scholarly writing and ethical publishing standards for nursing professionals and doctoral students in the AI era. Addresses the ethical challenges of AI-generated content in academic writing, providing practical guidance for maintaining scholarly integrity. |
| 104. | Nesreen Alqaissi & Mohammad Qtait | 2025 | Knowledge, attitudes, practices, and barriers regarding the integration of artificial intelligence in nursing and health sciences education: a systematic review | Systematic review | Multiple AI applications; knowledge, attitudes, practices, and barriers to AI integration in nursing and health sciences education | * Professional identity formation: Not addressed. * Ethical reasoning: Present — ethical attitudes toward AI. * Moral agency: Not addressed. * Clinical judgment: Not addressed. * Digital professionalism: Present — AI knowledge and competency. * Power awareness: Not addressed. * Recognition of clinical AI infrastructures: Not addressed. | Systematic review of knowledge, attitudes, practices, and barriers to AI integration in nursing and health sciences education. Finds knowledge gaps and attitudinal barriers are primary obstacles to AI integration; identifies need for structured AI education and institutional support to overcome barriers. |
| 105. | H Scroggins | N/A | Exploration of Academic Clinical Nurse Educators Moral Distress While Working with Students in the Clinical Setting: A Phenomenology Study | Empirical study (qualitative phenomenological) | AI in simulation/education; academic clinical nurse educators’ moral distress in clinical settings | * Professional identity formation: Present — clinical nurse educator identity and moral distress. * Ethical reasoning: Present — moral distress as ethical experience. * Moral agency: Present — central focus; nurse educator moral agency. * Clinical judgment: Present — clinical judgment in student supervision. * Digital professionalism: Not addressed. * Power awareness: Present — power dynamics in clinical education. * Recognition of clinical AI infrastructures: Not addressed. | Phenomenological study of academic clinical nurse educators’ moral distress while working with students in clinical settings. Identifies sources of moral distress including institutional constraints, student safety concerns, and role conflicts. Highly relevant to professional formation and moral agency in nursing education. |
| 106. | Walaa Badawy et al. | 2025 | Artificial intelligence in nursing practice and education: A 10-year bibliometric analysis of trends, gaps and emerging frontiers | Empirical study (bibliometric analysis) | Multiple AI applications; 10-year bibliometric analysis of AI in nursing practice and education | * Professional identity formation: Not addressed. * Ethical reasoning: Not addressed. * Moral agency: Not addressed. * Clinical judgment: Not addressed. * Digital professionalism: Not addressed. * Power awareness: Not addressed. * Recognition of clinical AI infrastructures: Not addressed. | 10-year bibliometric analysis of AI in nursing practice and education. Maps research trends, key themes, leading authors, and publication patterns. Identifies rapid growth in AI nursing research with education and clinical decision support as dominant themes, and highlights emerging frontiers. |
| 107. | Loutfy et al. | 2025 | Digital proficiency: a self-assessment of eHealth literacy and informatics competencies among nursing students. | Empirical study (cross-sectional/self-assessment) | Multiple AI applications; eHealth literacy and informatics competencies among nursing students | * Professional identity formation: Not addressed. * Ethical reasoning: Not addressed. * Moral agency: Not addressed. * Clinical judgment: Not addressed. * Digital professionalism: Present — eHealth literacy and informatics competency as digital professionalism. * Power awareness: Not addressed. * Recognition of clinical AI infrastructures: Not addressed. | Self-assessment study of eHealth literacy and informatics competencies among nursing students. Finds significant gaps in digital proficiency, particularly in informatics and eHealth literacy. Highlights need for structured digital competency education in nursing curricula. |
| 108. | Sürme et al. | 2026 | A new era in the education and practice of nurse academics: A qualitative study on the use of artificial intelligence. | Empirical study (qualitative) | Multiple AI applications; AI use by nurse academics — new era in education and practice | * Professional identity formation: Present — nurse academic identity in AI era. * Ethical reasoning: Present — ethical dimensions of AI use in academic practice. * Moral agency: Present — educator agency in AI adoption. * Clinical judgment: Not addressed. * Digital professionalism: Present — AI competency of nurse academics. * Power awareness: Not addressed. * Recognition of clinical AI infrastructures: Not addressed. | Qualitative study of nurse academics’ experiences with AI in a new era of education and practice. Identifies themes of adaptation, uncertainty, and professional identity renegotiation. Calls for institutional support and community of practice approaches to support nurse academics in navigating AI integration. |
| 109. | Fisher et al. | 2025 | Preparing Doctoral Nurse Leaders to Champion Artificial Intelligence in Nursing Education. | Commentary/editorial | Multiple AI applications; preparing doctoral nurse leaders to champion AI in nursing education | * Professional identity formation: Present — doctoral nurse leader identity and AI advocacy. * Ethical reasoning: Present — ethical AI leadership. * Moral agency: Present — nurse leader moral agency in AI governance. * Clinical judgment: Not addressed. * Digital professionalism: Present — AI leadership competency. * Power awareness: Present — leadership power in AI advocacy and governance. * Recognition of clinical AI infrastructures: Present — institutional AI systems. | Argues for preparing doctoral nurse leaders to champion AI integration in nursing education. Identifies key competencies for AI leadership including ethical governance, strategic advocacy, and change management. Positions doctoral nurses as critical agents in shaping responsible AI integration in nursing. |
| 110. | Martis et al. | 2025 | Enhancing Competencies of Nursing Students in Pain Management Education Through Artificial Intelligence (AI): A Narrative Review. | Narrative review | Multiple AI applications; AI for enhancing nursing students’ competencies in pain management education | * Professional identity formation: Not addressed. * Ethical reasoning: Present — ethical dimensions of pain management. * Moral agency: Present — nurse moral agency in pain management. * Clinical judgment: Present — pain assessment and management clinical judgment. * Digital professionalism: Present — AI tools for nursing education. * Power awareness: Not addressed. * Recognition of clinical AI infrastructures: Not addressed. | Narrative review of AI for enhancing nursing student competencies in pain management education. Identifies AI tools (simulation, decision support, adaptive learning) that can improve pain assessment and management competencies, with implications for clinical judgment development. |
| 111. | Shelly Amsberry | 2022 | Promoting academic integrity in nursing education: An integrative review | Integrative review | AI in simulation/education; academic integrity in nursing education (pre-AI era) | * Professional identity formation: Present — professional integrity as identity formation. * Ethical reasoning: Present — academic integrity as ethical practice. * Moral agency: Present — student moral agency in academic integrity. * Clinical judgment: Not addressed. * Digital professionalism: Not addressed. * Power awareness: Not addressed. * Recognition of clinical AI infrastructures: Not addressed. | Integrative review of promoting academic integrity in nursing education (2022, pre-generative AI era). Provides foundational framework for academic integrity in nursing education, highly relevant as context for understanding AI-era academic integrity challenges. |
| 112. | M MS | N/A | Potential of Artificial Intelligence in Improvement of the Clinical, Educational, Decision-Making, Information and Research Skills of Nurses. | Literature review | Multiple AI applications; AI potential in clinical, educational, decision-making, information, and research skills of nurses | * Professional identity formation: Not addressed. * Ethical reasoning: Not addressed. * Moral agency: Not addressed. * Clinical judgment: Present — clinical and decision-making skills. * Digital professionalism: Present — AI competency across nursing domains. * Power awareness: Not addressed. * Recognition of clinical AI infrastructures: Present — AI in clinical and research settings. | Literature review of AI’s potential for improving clinical, educational, decision-making, information, and research skills of nurses. Provides comprehensive overview of AI applications across nursing domains, identifying opportunities for AI-enhanced professional development. |
| 113. | Schaal et al. | 2025 | Self-assessed vs. reported digital competence among health students in Germany, Ukraine and Kazakhstan: a DigComp 2.2-based cross-sectional study. | Empirical study (cross-sectional survey, multi-country) | Multiple AI applications; digital competence among health students in Germany, Ukraine, and Kazakhstan | * Professional identity formation: Not addressed. * Ethical reasoning: Not addressed. * Moral agency: Not addressed. * Clinical judgment: Not addressed. * Digital professionalism: Present — digital competence as core outcome (DigComp 2.2 framework). * Power awareness: Not addressed. * Recognition of clinical AI infrastructures: Not addressed. | Cross-sectional study of self-assessed vs. reported digital competence among health students in Germany, Ukraine, and Kazakhstan using DigComp 2.2. Finds discrepancies between self-assessed and actual digital competence, with implications for AI literacy education in health professions programs. |
| 114. | Aydogan et al. | 2026 | The effect of role-playing and AI-based learning on nursing students’ knowledge of the nursing process and diagnostic accuracy: A quasi-experimental study. | Empirical study (quasi-experimental) | AI in simulation/education; role-playing and AI-based learning on nursing students’ knowledge and diagnostic accuracy | * Professional identity formation: Not addressed. * Ethical reasoning: Not addressed. * Moral agency: Not addressed. * Clinical judgment: Present — diagnostic accuracy and nursing process knowledge. * Digital professionalism: Present — AI-based learning tools. * Power awareness: Not addressed. * Recognition of clinical AI infrastructures: Not addressed. | Quasi-experimental study of role-playing combined with AI-based learning on nursing students’ knowledge of the nursing process and diagnostic accuracy. Finds significant improvements in both outcomes in the AI-enhanced role-playing group, supporting AI integration in clinical skills education. |
| 115. | SMS ALOTAIBI & MS ALHELALI | N/A | Artificial Intelligence In Nursing Care Planning And Decision-Making: Current Applications And Challenges | Literature review | Multiple AI applications; AI in nursing care planning and decision-making — applications and challenges | * Professional identity formation: Not addressed. * Ethical reasoning: Present — ethical AI in clinical decision-making. * Moral agency: Not addressed. * Clinical judgment: Present — AI in care planning and clinical decision-making. * Digital professionalism: Present — AI competency for clinical nurses. * Power awareness: Not addressed. * Recognition of clinical AI infrastructures: Present — AI systems in care planning. | Reviews current AI applications in nursing care planning and decision-making. Identifies AI tools for care plan generation, clinical decision support, and patient monitoring. Discusses challenges including accuracy concerns, accountability questions, and the risk of displacing nursing clinical judgment. |
| 116. | CN Anazia | N/A | THE ETHICAL DIMENSIONS OF ARTIFICIAL INTELLIGENCE IN NURSING PRACTICE: BALANCING INNOVATION AND HUMAN CARE | Commentary/editorial | AI ethics/bias; ethical dimensions of AI in nursing practice — balancing innovation and human care | * Professional identity formation: Present — nursing humanistic identity in AI era. * Ethical reasoning: Present — central focus; ethical AI in nursing practice. * Moral agency: Present — nurse moral agency in AI-integrated practice. * Clinical judgment: Present — human clinical judgment vs. AI. * Digital professionalism: Present — professional standards for AI in nursing. * Power awareness: Present — power dynamics of AI in healthcare. * Recognition of clinical AI infrastructures: Present — AI systems in clinical nursing. | Critical commentary on the ethical dimensions of AI in nursing practice. Argues for a humanistic approach to AI integration that preserves nursing’s core values of compassion, advocacy, and ethical care. Calls for nursing leadership in shaping ethical AI governance frameworks. |
| 117. | Passwater & Passwater | 2025 | Educating With Edge: Aligning Bloom’s Revised Taxonomy With the Substitution, Augmentation, Modification, and Redefinition Model to Enhance Graduate Nursing Education for the Artificial Intelligence Era. | Commentary/editorial | Multiple AI applications; aligning Bloom’s Taxonomy with SAMR model for graduate nursing education in AI era | * Professional identity formation: Not addressed. * Ethical reasoning: Not addressed. * Moral agency: Not addressed. * Clinical judgment: Not addressed. * Digital professionalism: Present — AI-era pedagogical framework for graduate nursing. * Power awareness: Not addressed. * Recognition of clinical AI infrastructures: Not addressed. | Proposes aligning Bloom’s Revised Taxonomy with the SAMR model to enhance graduate nursing education for the AI era. Provides a practical framework for designing AI-integrated learning experiences that develop higher-order thinking. |
| 118. | Campbell & Tiase | 2026 | Bridging the Gap Between Potential and Practice: An Integrative Review of Generative Artificial Intelligence in Nursing. | Integrative review | ChatGPT/generative AI; bridging gap between potential and practice of generative AI in nursing | * Professional identity formation: Present — nursing professional identity in generative AI era. * Ethical reasoning: Present — ethical dimensions of generative AI in nursing. * Moral agency: Present — nurse agency in AI-integrated practice. * Clinical judgment: Present — AI and nursing clinical decision-making. * Digital professionalism: Present — professional standards for generative AI in nursing. * Power awareness: Present — power dynamics of AI adoption in nursing. * Recognition of clinical AI infrastructures: Present — generative AI as nursing practice infrastructure. | Integrative review bridging the gap between the potential and practice of generative AI in nursing. Identifies the promise of generative AI for nursing education and clinical practice, alongside significant implementation barriers including ethical concerns, workforce unpreparedness, and lack of governance frameworks. |
| 119. | Ryan Yung et al. | 2024 | Mitigating the impact of ChatGPT: Cultivating professional identity through interactive oral authentic assessments | Empirical study (quasi-experimental/descriptive) | ChatGPT/generative AI; mitigating ChatGPT impact through interactive oral authentic assessments | * Professional identity formation: Present — professional identity cultivation through authentic assessment. * Ethical reasoning: Present — academic integrity as ethical practice. * Moral agency: Present — student moral agency and responsibility. * Clinical judgment: Not addressed. * Digital professionalism: Present — responsible AI use and academic integrity. * Power awareness: Not addressed. * Recognition of clinical AI infrastructures: Not addressed. | Examines mitigating ChatGPT’s impact on academic integrity through interactive oral authentic assessments. Finds oral assessments effectively reduce AI-assisted dishonesty while cultivating professional identity, communication skills, and moral agency. Proposes authentic assessment as a key strategy for AI-era nursing education. |
